# VRK1 Phosphorylates Tip60/KAT5 and Is Required for H4K16 Acetylation in Response to DNA Damage

**DOI:** 10.3390/cancers12102986

**Published:** 2020-10-15

**Authors:** Raúl García-González, Patricia Morejón-García, Ignacio Campillo-Marcos, Marcella Salzano, Pedro A. Lazo

**Affiliations:** 1 Molecular Mechanisms of Cancer Program, Instituto de Biología Molecular y Celular del Cáncer, CSIC-Universidad de Salamanca, Campus Miguel de Unamuno, 37007 Salamanca, Spain; garciaraul@usal.es (R.G.-G.); pmoreg@usal.es (P.M.-G.); ignacio_cm@usal.es (I.C.-M.); 2 Área de Cancer, Instituto de Investigación Biomédica de Salamanca-IBSAL, Hospital Universitario de Salamanca, 37007 Salamanca, Spain; 3 Enfermedades Digestivas y Hepáticas, Vall d’Hebron Institut de Recerca, Hospital Universitari Vall d’Hebron, Universidad Autónoma de Barcelona, 08035 Barcelona, Spain; marcella.salzano@vhir.org

**Keywords:** phosphorylation, histone H4, acetylation, DNA-damage response, nucleosomal histone kinase-1

## Abstract

**Simple Summary:**

Dynamic remodeling of chromatin requires epigenetic modifications of histones. DNA damage induced by doxorubicin causes an increase in histone H4K16ac, a marker of local chromatin relaxation. We studied the role that VRK1, a chromatin kinase activated by DNA damage, plays in this early step. VRK1 depletion or MG149, a Tip60/KAT5 inhibitor, cause a loss of H4K16ac. DNA damage induces the phosphorylation of Tip60 mediated by VRK1 in the chromatin fraction. VRK1 directly interacts and phosphorylates Tip60. This phosphorylation of Tip60 is lost by depletion of VRK1 in both *ATM* +/+ and *ATM*−/− cells. Kinase-active VRK1, but not kinase-dead VRK1, rescues Tip60 phosphorylation induced by DNA damage independently of ATM. The VRK1 chromatin kinase is an upstream regulator of the initial acetylation of histones, and an early step in DNA damage responses.

**Abstract:**

Dynamic remodeling of chromatin requires acetylation and methylation of histones, frequently affecting the same lysine residue. These alternative epigenetic modifications require the coordination of enzymes, writers and erasers, mediating them such as acetylases and deacetylases. In cells in G0/G1, DNA damage induced by doxorubicin causes an increase in histone H4K16ac, a marker of chromatin relaxation. In this context, we studied the role that VRK1, a chromatin kinase activated by DNA damage, plays in this early step. VRK1 depletion or MG149, a Tip60/KAT5 inhibitor, cause a loss of H4K16ac. DNA damage induces the phosphorylation of Tip60 mediated by VRK1 in the chromatin fraction. VRK1 directly interacts with and phosphorylates Tip60. Furthermore, the phosphorylation of Tip60 induced by doxorubicin is lost by depletion of VRK1 in both *ATM* +/+ and *ATM*−/− cells. Kinase-active VRK1, but not kinase-dead VRK1, rescues Tip60 phosphorylation induced by DNA damage independently of ATM. The Tip60 phosphorylation by VRK1 is necessary for the activating acetylation of ATM, and subsequent ATM autophosphorylation, and both are lost by VRK1 depletion. These results support that the VRK1 chromatin kinase is an upstream regulator of the initial acetylation of histones, and an early step in DNA damage responses (DDR).

## 1. Introduction

Chromatin remodeling underlines all biological processes of the genome. The dynamic reorganization of chromatin requires several different, or alternative, epigenetic modifications of histones, which include acetylation, methylation, ubiquitination, and phosphorylation. Different combinations of these epigenetic marks are associated to the compaction or relaxation of chromatin and its biological functions including transcription, replication, differentiation, telomere protection, genome stability and DNA damage repair (DDR) [1,2,3,4]. Each individual cell has to remodel its chromatin to adjust to its particular functional situation. The pattern of histone modifications determines the tumor epigenome in tumor cells [5,6], which is also reflected in the tumor cell heterogeneity [7]. Some of these alternative epigenetic modifications frequently affect the same lysine residue in the targeted histone [8,9,10], such as acetylations and methylations, and there is crosstalk among these different histone modifications [2,4].

This dynamic chromatin remodeling involves at least four different enzyme families with several members, which includes writers such as lysine acetyl transferases (KAT) and methyl transferases (KMT), as well as erasers such as lysine deacetylases (HDAC) and demethylases (KDM) [2,4,11]. All of them are active target for pharmacological development in cancer [3,12]. The transition from one epigenetic histone modification to another requires the coordination of two, or four, of these enzymes, depending on the types of modifications in a specific lysine residue. However, the coordinators of these alternative enzymes and their functional and temporal organization are unknown.

Kinases are likely candidates to regulate, or coordinate, the balance of alternative histone epigenetic modifications, depending on the individual cell functional needs, by phosphorylation in Ser or Thr residues in nucleosomal histones [3], as well as by the regulation of the enzymes implicated in these modifications. Among them, human VRK1 (Q99986) [13], also known as nucleosomal-histone kinase 1 (NHK1) in *Drosophila melanogaster* [14], a very abundant nuclear/chromatin kinase [15], is well situated to participate in these processes. Human VRK1 is involved in the regulation of cell proliferation [16,17,18], and DNA damage responses [19,20,21,22]. VRK1 also specifically phosphorylates and regulates several transcription factors such as p53 [23,24,25], c-Jun [26], ATF2 [27], CREB [28], and Sox2 [18]. Furthermore, VRK1 also phosphorylates BAF, which is involved in regulation of the nuclear envelope assembly and chromosome attachment, and their disruption cause DNA chromosomal alterations [29,30,31]. The kinase activity of VRK1 is induced by different types of DNA damage able to cause single or double strand breaks or alkylating lesions [19]. In response to DNA damage, VRK1 directly phosphorylates histones H3 [21,32,33,34], H2AX [14,35], NBS1 [20], and 53BP1 [19,36]. VRK1 also phosphorylates hnRNPA1 in telomerase activation [37]. The VRK1 kinase activity is regulated by interactions of its C-terminal regulatory region with different proteins [38], such as the macroH2A histone variant [18,39], or with nuclear Ran [40].

When DNA damage occurs, each individual cell has to respond independently of its particular situation regarding resting or dividing, differentiation stage and the heterogeneity of its cellular or protein interactions. The initial response to DNA damage implicates a local chromatin relaxation that is associated to histone H4 acetylation in K16 [41,42,43]. Therefore, H4K16 hypo-acetylation is associated with defective DNA repair [36,44]. The importance of H4K16ac in order to trigger a proper DNA-damage response in a locally altered chromatin indicates that this modification is tightly regulated. However, the regulation of histone H4 acetylation has to be indirect and mediated by regulation of the Tip60/KAT5 (Q92993) acetylase, as a potential candidate. The acetylation in K5 of H2AX by Tip60 is necessary for the recruitment of NBS1 to damaged chromatin [45]. Tip60 also regulates chromatin recognition by 53BP1 [46]. Impairment of H4K16ac interferes with the docking site of NBS1, which is required for recruitment of 53BP1 in DNA damage responses [47]. VRK1 depletion impairs both NBS1 [20] and 53BP1 [19] specific phosphorylation and activation in response to DNA damage. Moreover, Tip60 is necessary for the acetylation and activation by autophosphorylation of ATM in response to DNA damage [48], whereas VRK1 is also essential for ATM activation [19,20,36]. Overexpression of Tip60 is associated to a poorer prognosis [49] and to cisplatin resistance in tumor cells [50]. High levels of VRK1 are also associated to poor prognosis [51] and confer resistance to DNA-damage based treatments [52]. All these data place VRK1 as a candidate to regulate Tip60 activity and H4K16ac.

In this work, we have studied the role that the VRK1 chromatin kinase played in the regulation of Tip60/KAT5, and its association to both very early upstream events in DNA-damage response, such as H4K16 acetylation (responsible for a local chromatin relaxation), and sequential steps in the context of the ATM pathway during DNA damage response.

## 2. Results

### 2.1. DNA Damage Induces Phosphorylation of Tip60 and Acetylation of H4K16

**Figure 1:**
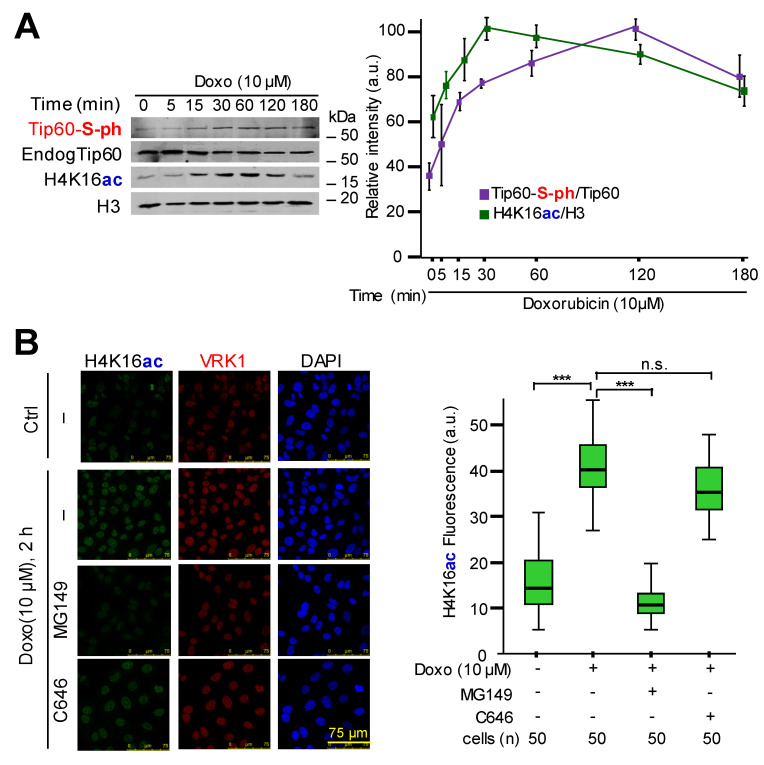
Effect of KAT (Lysine acetyl transferase) inhibitors on the acetylation of histone H4 induced by doxorubicin. (**A**). Time course of the effect of doxorubicin on the phosphorylation of endogenous Tip60 in serine detected with anti-phosphoserine antibody, and the acetylation of histone H4 in K16 in cells deprived of serum (0.5%) for 48 h and arrested in G0/G1 before treatment with doxorubicin (Doxo). Histones were prepared by acidic extraction. The reference value corresponds to the starting time point (0 min). (**B**). Induction of H4K16ac by treatment with doxorubicin (10 µM) and the effect of MG149 (Tip60 inhibitor, 2 µM) and C646 (p300 inhibitor, 10 µM) added ten hours before doxorubicin addition. The box plots show the quantification of the fluorescence using the Image J program. ns: Not significant. *** *p* < 0.001.

To address the role of human VRK1 in the chromatin relaxation associated to histone acetylation, it was determined whether the activation of Tip60 by doxorubicin, leading to the acetylation of H4K16, might be associated to Tip60 phosphorylation by a not yet identified kinase. To eliminate mitogenic signals that activate VRK1 [17], cells were serum-deprived for 48 h. In these conditions, cells accumulated in G0/G1, there is no expression of cyclinD1, and retinoblastoma is not phosphorylated [17]. The induction of DNA damage by doxorubicin was confirmed by determination of free DNA ends in TUNEL assays (Figure S1A), and the DDR was confirmed by determining the formation of 53BP1 foci induced by double–strand breaks caused by doxorubicin, a response that is prevented by VRK1 knockdown (Figure S1B). These cells were treated with doxorubicin and they responded with an increase in the phosphorylation of endogenous Tip60 (Figure 1A), as well as an increase in H4K16ac (Figure 1A). To confirm the role of Tip60, we tested the effect of two HAT inhibitors that target HATs associated to two different roles of VRK1, DDR, and transcription. The inhibitor MG149 that targets Tip60 [53] prevented H4K16 acetylation in response to DNA damage induced by doxorubicin, but this acetylation was not affected by C646 that inhibits p300 [54], in serum-deprived A549 cells (Figure 1B) and U2OS cells (Figure S2).

### 2.2. Kinase-Active VRK1 Rescues H4K16 Acetylation Induced by DNA Damage

**Figure 2:**
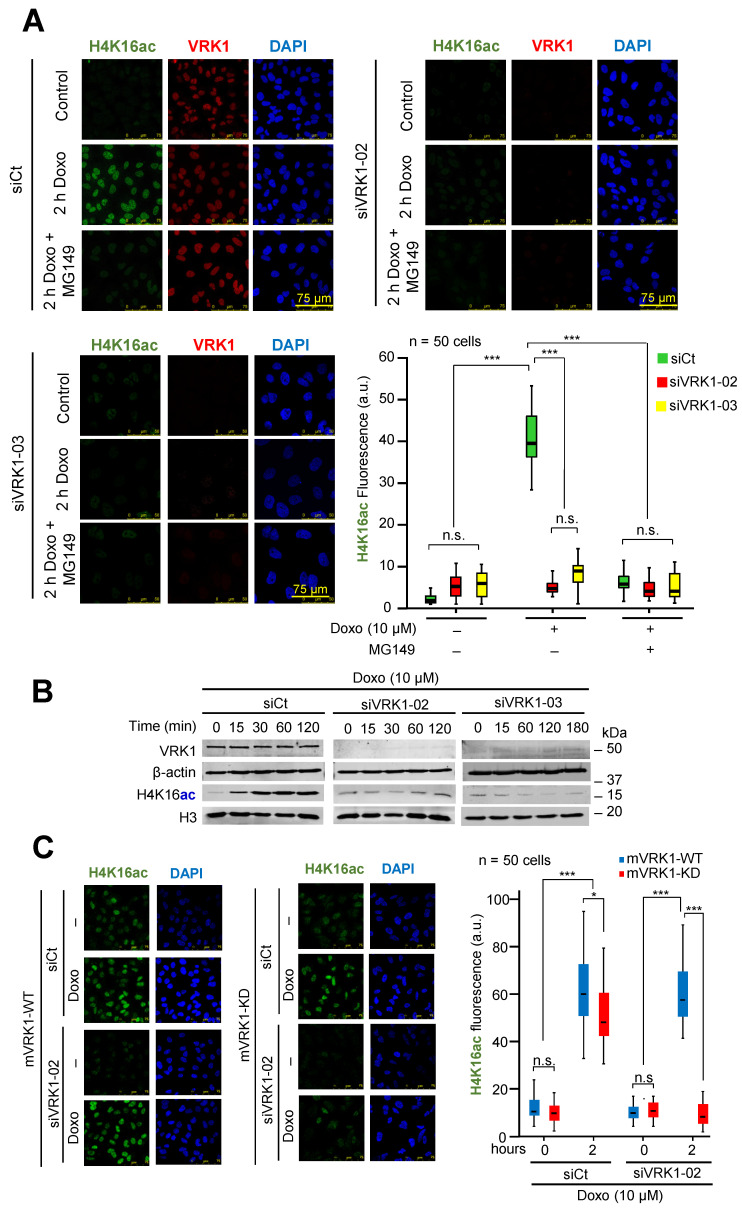
Effect of VRK1 depletion on H4K16 acetylation. (**A**). VRK1 was knockdown in A549 cells using siVRK1-02 (siV-02) and siVRK1-03 (siV-03). Serum-deprived (0.5% FBS for 48 h) cells were treated with 10 µM doxorubicin (Doxo) for 3 h to induce DNA damage. MG149 (2 µM) was added to the cell culture at 12 h before doxorubicin. ns: Not significant, *** *p* < 0.001. (**B**). Effect of VRK1 depletion with two different siRNA on endogenous H4K16 acetylation induced by doxorubicin (Doxo) and detected by immunoblot. (**C**). Rescue of H4K16ac induced by doxorubicin in siVRK1-02 depleted cells and their rescue with either murine VRK1 or kinase-dead VRK1-KD (K179E) in stable A549 cell lines. All experiments were repeated three times. siCt: siControl. *** *p* < 0.001, * *p* < 0.1.

Depletion of VRK1 caused a loss of H4K16ac in cells that were serum-deprived to prevent mitogenic activation of VRK1 [21,34]. Therefore, we hypothesized that VRK1 chromatin kinase might be a candidate kinase to regulate the acetylation of histone H4. The regulation of Tip60/KAT5 by a VRK1-mediated phosphorylation is a likely mechanism that regulates this acetylation. Therefore, to determine whether VRK1 can be a candidate kinase regulating Tip60 phosphorylation, its depletion should have a similar effect to that of Tip60 inhibitors on H4K16 acetylation. For this aim, it was determined whether H4K16ac induced by DNA damage and caused by doxorubicin [21], was sensitive to VRK1 depletion, and to MG149, an inhibitor of the histone acetylase Tip60. Depletion of VRK1 by two different siRNA caused a loss of H4K16ac induced by doxorubicin, and this effect mimicked that caused by MG149 and detected by immunofluorescence (Figure 2A). The effect of VRK1 depletion on the loss of H4K16ac level induced by doxorubicin was also confirmed using two different siRNA in Western blots (Figure 2B). This effect was confirmed using a different cell line, U2OS cells, which have a higher basal level of histone acetylation in the absence of doxorubicin treatment. In U2OS cells, depletion of VRK1, and the MG149 Tip60 inhibitor, had a similar effect and resulted in a significant reduction of H4K16ac (Figure S2).

To confirm that the H4K16ac induced by doxorubicin was dependent on the kinase activity of VRK1, we performed a rescue experiment. For this aim, endogenous human VRK1 was depleted in stable A549 cells expressing either murine VRK1 (mVRK1) wild-type or kinase-dead (K179E) [19,55]. Following depletion of the endogenous human VRK1, only wild-type mVRK1, but not kinase-dead mVRK1-K179E, was able to rescue H4K16ac induced by doxorubicin (Figure 2C).

### 2.3. VRK1 Directly Interacts with and Phosphorylates Tip60

**Figure 3:**
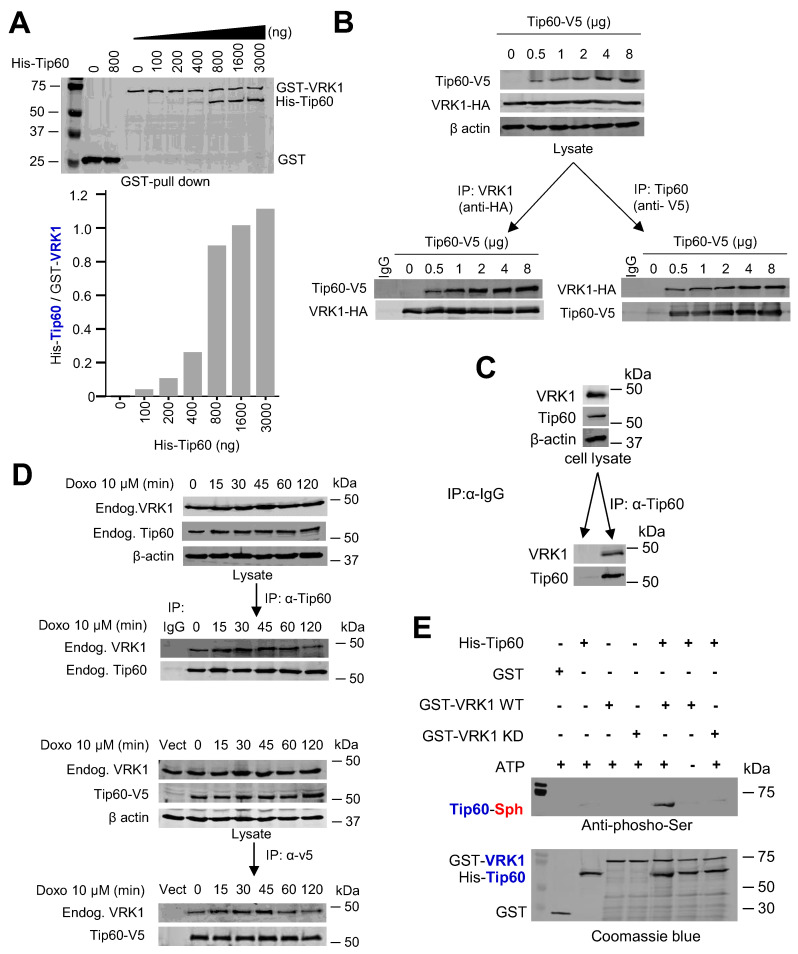
VRK1 direct interaction and phosphorylation of Tip60. (**A**). Binding of Tip60 to human VRK1. Increasing amounts of His-Tip60 were incubated with a fixed amount (1000 ng) of GST-VRK1, followed by a pulldown of GST-VRK1 and determination of Tip60. In the gel was included an assay with GST to rule out a non-specific interaction with His-Tip60. Proteins were detected by immunoblot with specific monoclonal antibodies for the GST and His epitopes. (**B**). Direct interaction between VRK1 and Tip60. Cells were transfected with tagged plasmids expressing human VRK1-HA and Tip60-V5. The complex between these two proteins within cells was detected by reciprocal immunoprecipitations. (**C**). Immunoprecipitation of endogenous Tip60 with an anti-Tip60 antibody (ab137518) and detection of endogenous VRK1 in the immunoprecipitate. (**D**). Doxorubicin treatment does not alter the interaction between endogenous VRK1 and Tip60 (top) or between endogenous VRK1 and transfected Tip60-V5 (bottom). (**E**). Direct in vitro phosphorylation of Tip60 by VRK1 using bacterially expressed and purified proteins. Phosphorylated Tip60 was determined with an anti-phosphoserine antibody.

To test whether VRK1 and Tip60 are directly related, we performed two types of assays with VRK1 and Tip60 human proteins. First, we performed an interaction assay from proteins expressed in bacteria and purified. In this experiment, a fixed amount of GST-VRK1 was incubated with increasing amounts of His-Tip60. The GST-VRK1 pulldown detected an increase in its interaction with His-Tip60 as the concentration of the latter was higher, and reaching a plateau as their concentrations became equimolar (Figure 3A). This indicated that both proteins are able to form a direct stable complex by themselves. To confirm the interaction in vivo, A549 cells were transfected with Tip60-V5 and VRK1-HA tagged human proteins, and a reciprocal immunoprecipitation was performed. The Tip60-VRK1 complex was detected in both reciprocal precipitations indicating it is stable by itself (Figure 3B). To confirm this interaction, the endogenous Tip60 was immunoprecipitated and the endogenous VRK1 detected in the immunoprecipitate (Figure 3C). In addition, we determined that the interaction between endogenous VRK1 and endogenous or transfected Tip60 was not affected by DNA damage (Figure 3D).

To demonstrate that the phosphorylation of Tip60 by VRK1 is also direct, we performed an in vitro kinase assay, with bacterially expressed and purified proteins. The assay was done with either active VRK1, or kinase-dead VRK1 (K179E) as negative control [19,40,55], and His-Tip60 as substrate. The phosphorylation of Tip60 was determined by immunoblot. Tip60 was phosphorylated by kinase-active VRK1, but not by kinase-dead VRK1 (K179E) (Figure 3E). Therefore, we concluded that VRK1 directly phosphorylates Tip60.

### 2.4. Kinase-Active VRK1 Mediates the Phosphorylation of Tip60 Induced by DNA-Damage

**Figure 4:**
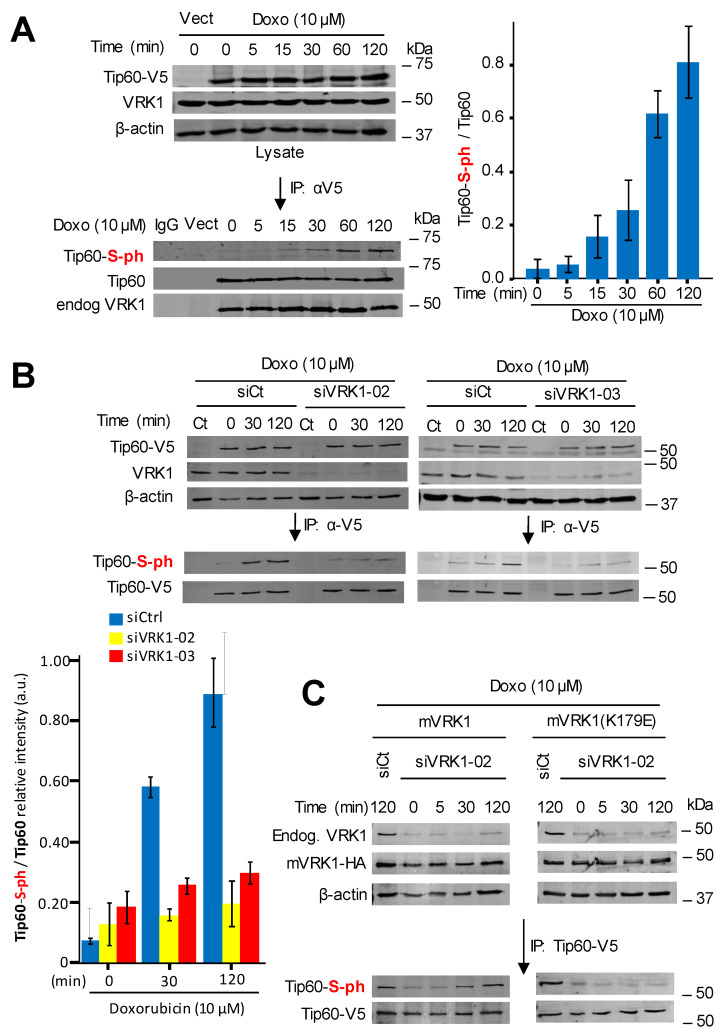
VRK1 mediates the phosphorylation of Tip60 induced by doxorubicin. (**A**). Time dependent phosphorylation of Tip60 in cells treated with doxorubicin. A549 cells were transfected with plasmid Tip60-V5 or empty vector (Ct); and to eliminate mitogenic signaling, cells were maintained in 0.5% serum for 48 h before the addition of doxorubicin to the culture. Cells were lysed at different time points and Tip60 was immunoprecipitated and determined its stable interaction with endogenous VRK1. The phosphorylation of the Tip60 after doxorubicin treatment and present in the immunoprecipitated Tip60-VRK1 complex was detected in Western blots. To the right it is shown the quantification from three independent experiments. Vect: Transfected with empty vector. Vect: Transfection control with empty vector. (**B**). Effect of VRK1 depletion on the phosphorylation of Tip60. A549 cells were treated with si-control (left), siVRK1-02 (center) or siVRK1-03 (right) followed by transfection with plasmid Tip60-V5, and cells were treated with doxorubicin at the indicated times. Transfected Tip60-V5 was immunoprecipitated and the phosphorylation of Tip60 determined in Western blots. The graph to the bottom-left shows the quantification of phosphorylated Tip60 from three different experiments. Vect: Transfected with empty vector. (**C**). Rescue of Tip60 phosphorylation induced by doxorubicin in two A549 stable cell lines expressing either murine kinase-active (left) or kinase-dead murine VRK1 (K179E) (right) in which endogenous human VRK1 was depleted. Experiments were repeated three times.

It is known that the kinase activity of VRK1 is induced by DNA damage independently of its type, including damage caused by doxorubicin [19]. Therefore, we tested whether incubation of A549 cells with doxorubicin was able to cause the phosphorylation of Tip60 in cells treated with doxorubicin. The treatment with doxorubicin did not alter the intracellular complex formed between the endogenous VRK1 and the transfected Tip60 determined by immunoprecipitation (Figure 4A). However, doxorubicin treatment induced an increase in the level of phosphorylated-Tip60 that is complexed with VRK1 (Figure 4A). Next, it was determined whether this Tip60 phosphorylation induced by doxorubicin was dependent on VRK1. For this aim, endogenous human VRK1 was depleted using two different siRNAs and the level of Tip60 phosphorylation induced by doxorubicin was determined in the immunoprecipitate. The increase of Tip60 phosphorylation induced by doxorubicin was lost in VRK1-depleted cells by using two different siRNA (Figure 4B).

Next, we performed a rescue experiment to confirm the effect of VRK1 on the phosphorylation of Tip60 in response to doxorubicin treatment. For this purpose, stable A549 cell lines were made, one expressing wild-type murine VRK1 (mVRK1), and the other by expressing kinase-dead murine VRK1-(K179E) [55]. In these cells, the effect of the murine VRK1 proteins was determined after depletion of the endogenous human VRK1. Wild-type murine VRK1 was able to rescue the phosphorylation of Tip60 induced by doxorubicin, but the kinase-dead mVRK1-(K179E) did not rescue Tip60 phosphorylation (Figure 4C).

### 2.5. VRK1 Depletion Prevents the Accumulation in Chromatin of Phospho-Tip60 Induced by DNA Damage

**Figure 5:**
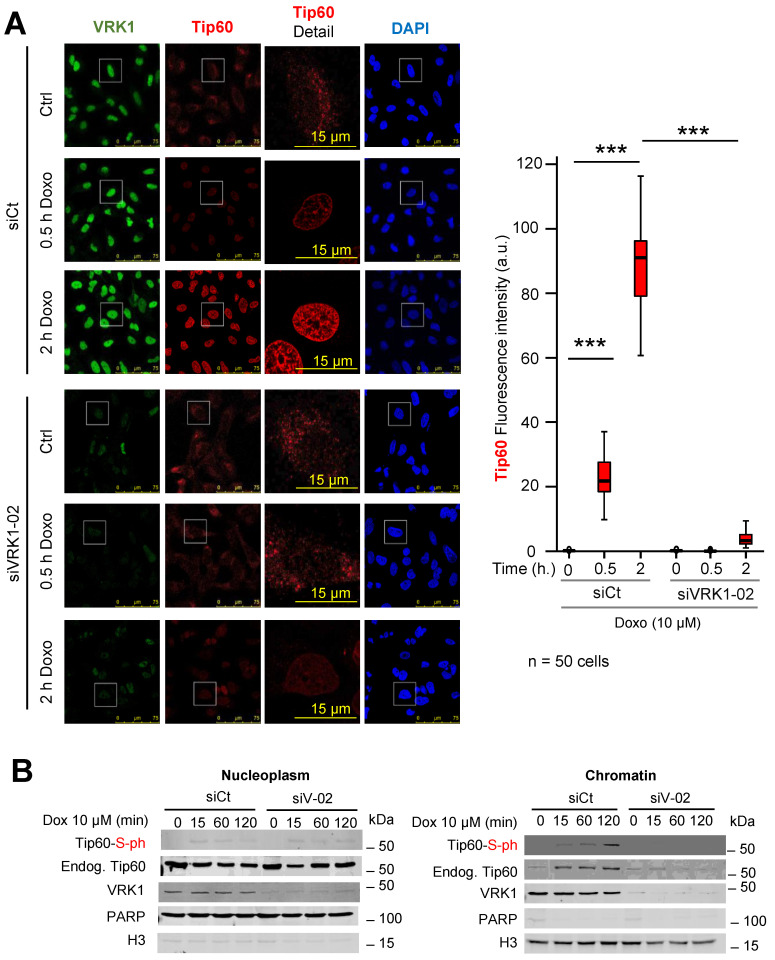
VRK1 knockdown prevents chromatin accumulation of endogenous Tip60 induced by doxorubicin. (**A**). Effect of doxorubicin on the Tip60 nuclear protein detected by immunofluorescence. siCt: si-control; si-V1-02: si-VRK1-02. The fluorescence was quantified in fifty cells using the Image J program. *** *p* < 0.001. (**B**). Effect of doxorubicin on the endogenous Tip60 and its phosphorylation in the nucleoplasm and chromatin fractions determined in control or VRK1-depleted A549 cells. In all time course experiments, the reference value is the one corresponding to the starting time point (0 min). All experiments were repeated four times.

VRK1 is a nuclear kinase present in nucleoplasm and chromatin [21,32]. Therefore, it was determined whether the effect of VRK1 depletion on the nuclear localization of endogenous Tip60/KAT5 protein was dependent on its localization within nuclei. VRK1 was depleted with siRNA, and cells were treated with doxorubicin. In control cells, doxorubicin induced a significant accumulation of endogenous Tip60 in nuclei in response to DNA damage (Figure 5A). In VRK1 depleted cells, there was a loss of nuclear fluorescence associated to endogenous Tip60 (Figure 5A).

Next, it was determined whether the phosphorylated endogenous Tip60 was associated to the chromatin fraction of the nuclear protein. For this aim, the localization of phosphorylated Tip60 in response to doxorubicin was determined by fractionation of nuclei into chromatin and nucleoplasm fractions. The presence of phosphorylated endogenous Tip60 induced by doxorubicin was only detected in the chromatin fraction (Figure 5B right), but not in the nucleoplasm (Figure 5B left). Furthermore, this chromatin localization of endogenous Tip60 was lost by depletion of VRK1 (Figure 5B right). These data indicated that the phosphorylation of Tip60 occurred on chromatin where VRK1 is also present, but not in the nucleoplasm fraction.

### 2.6. The Phosphorylation of Tip60 by VRK1 is Induced by Doxorubicin in ATM Null Cells

**Figure 6:**
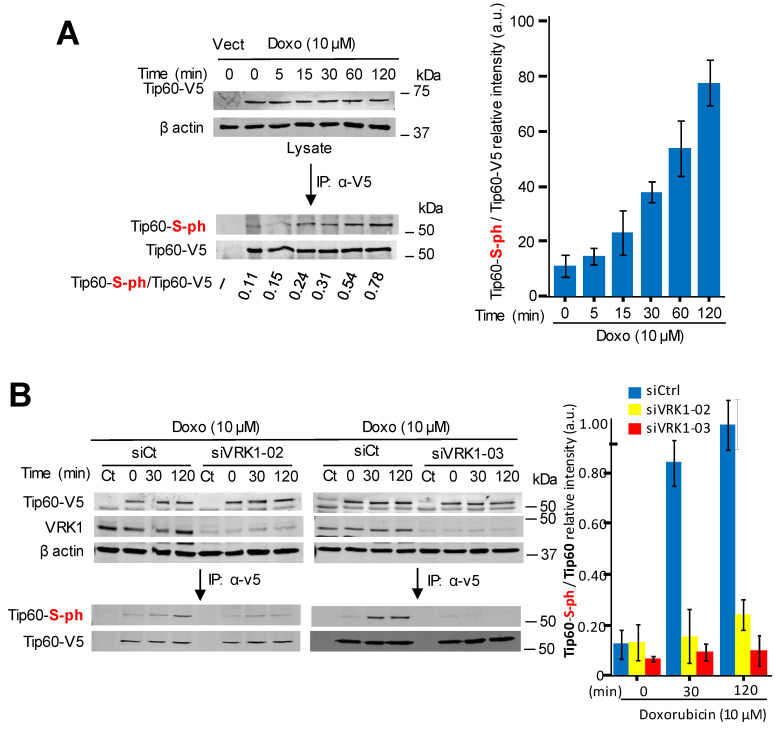
The phosphorylation of Tip60 is independent of ATM and lost by VRK1 depletion. (**A**). Time course of the phosphorylation of Tip60 by DNA damage induced by doxorubicin in HT144 cells (*ATM*−/−) deprived of serum (0.5% FBS) for 48 h before treatment. Tip60 was immunoprecipitated and its phosphorylation determined in Western blots. In the graph to the right is shown the relative increase in Tip60 phosphorylation from three independent experiments. Ct is a negative control of the transfection with an empty vector. (**B**). Depletion of VRK1 with two different siRNA causes a loss of Tip60 phosphorylation induced by doxorubicin in HT144 cells (*ATM*−/−). To the right is shown the quantification of the relative phosphorylation in siControl and siVRK1 in HT144 cells (*ATM*−/−). The graph to the right shows the relative increase in Tip60 phosphorylation from three independent experiments. Ct: Cells transfected with empty vector.

The activation of ATM in DDR requires its previous acetylation in K3016 by Tip60 that induces its autophosphorylation in Ser1981 required for its kinase activity [56,57]. Moreover, the knockdown of VRK1 prevented the autophosphorylation of ATM in Ser1981 in DDR [19,20]. To rule out the possibility that the effect of VRK1 on Tip60 might be indirect and due to ATM, the induction of Tip60 phosphorylation in response to doxorubicin treatment was determined in the HT144 (*ATM−/−*) cell line. In these *ATM* deficient cells, doxorubicin induced the phosphorylation of Tip60 (Figure 6A), which indicated that ATM is not necessary for Tip60 phosphorylation. Next, we determined whether the Tip60 phosphorylation induced by doxorubicin in HT144 (*ATM*−/−) cells was also dependent on endogenous VRK1. The knockdown of VRK1 with two different siRNAs in HT144 (*ATM*−/−) cells resulted in the loss of Tip60 phosphorylation induced by doxorubicin (Figure 6B). This led to the conclusion that ATM does not mediate the phosphorylation of Tip60, and VRK1 is an upstream component in the pathway.

### 2.7. ATM Acetylation by Tip60 Is Insensitive to ATM Kinase Inhibitors but Is Lost by VRK1 Depletion or Tip60 Inhibition

**Figure 7:**
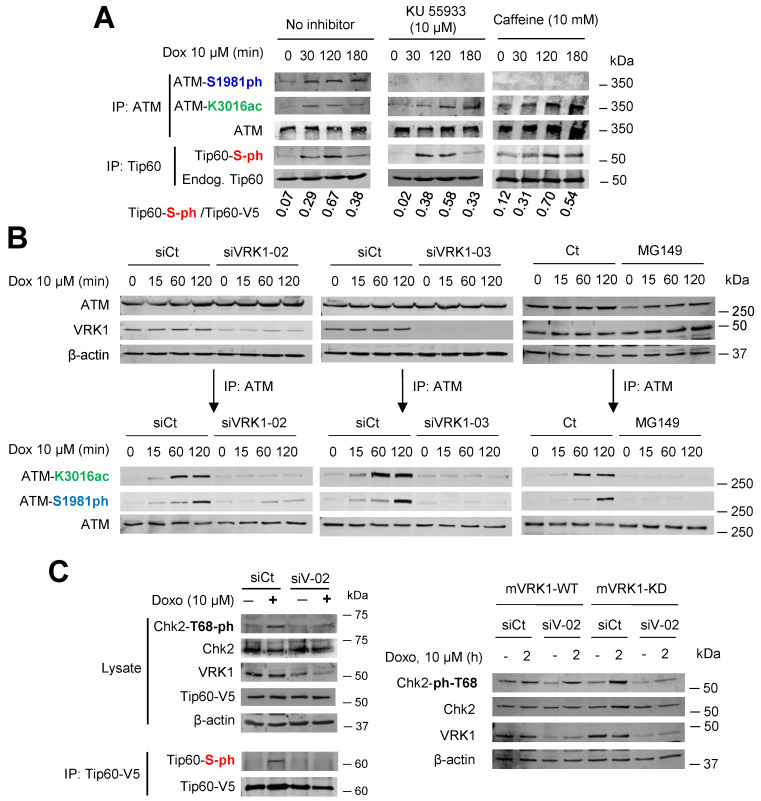
VRK1 is required for the acetylation of ATM by Tip60 in response to DNA damage induced by doxorubicin. (**A**). The phosphorylation and acetylation of ATM, and the phosphorylation of endogenous Tip60 were determined in A549 cells (ATM+/+) deprived of serum (0.5% FBS) for 48 h and treated with doxorubicin in combination with KU55933 or caffeine that inhibit ATM. The covalent modifications were detected in the immunoprecipitated endogenous proteins with specific antibodies. (**B**). Effect of VRK1 depletion with two different siRNA, and Tip60 inhibition with MG149, on the phosphorylation and acetylation of ATM in response to doxorubicin treatment in A549 cells. Treatment with the MG149 inhibitor was performed by its addition to cultures 12 h before cell lysis. (**C**). Effect of VRK1 depletion on the phosphorylation of CHK2 in T68, a downstream phosphorylation target of ATM in response to doxorubicin.

Next, we used a different approach to demonstrate that the phosphorylation of Tip60 is independent of ATM kinase activity. Serum deprived A549 (*ATM +/+*) cells were treated with KU55933, an inhibitor of ATM [58,59], or caffeine that inhibits ATM/ATR activity [60,61]. In the absence of these inhibitors, and in response to doxorubicin treatment, there was acetylation of ATM in K3016 [56], and phosphorylation of Ser1981 in ATM (Figure 7A, left), as positive control, as well as the predicted phosphorylation of Tip60. ATM forms an inactive dimer and its acetylation in K3016 by Tip60 leads to ATM autophosphorylation in Ser1981 [57]. However, the addition of KU55933 or caffeine inhibited the activating autophosphorylation of ATM in Ser1981 without affecting ATM acetylation in K3016 or the phosphorylation of Tip60 (Figure 7A, center and right). To demonstrate that ATM modifications, acetylation and phosphorylation, required VRK1, a different approach was used. Following depletion of endogenous VRK1 with two different siRNA, their effect on ATM-K3016 acetylation and ATM-S1981 phosphorylation induced by doxorubicin was determined in the immunoprecipitated endogenous ATM. Depletion of VRK1 resulted in the loss of both ATM acetylation and autophosphorylation (Figure 7B). Additionally, determining the phosphorylation of CHK2 in T68, a downstream target of ATM, which was also lost by VRK1 depletion, confirmed this observation (Figure 7C).

**Figure 8:**
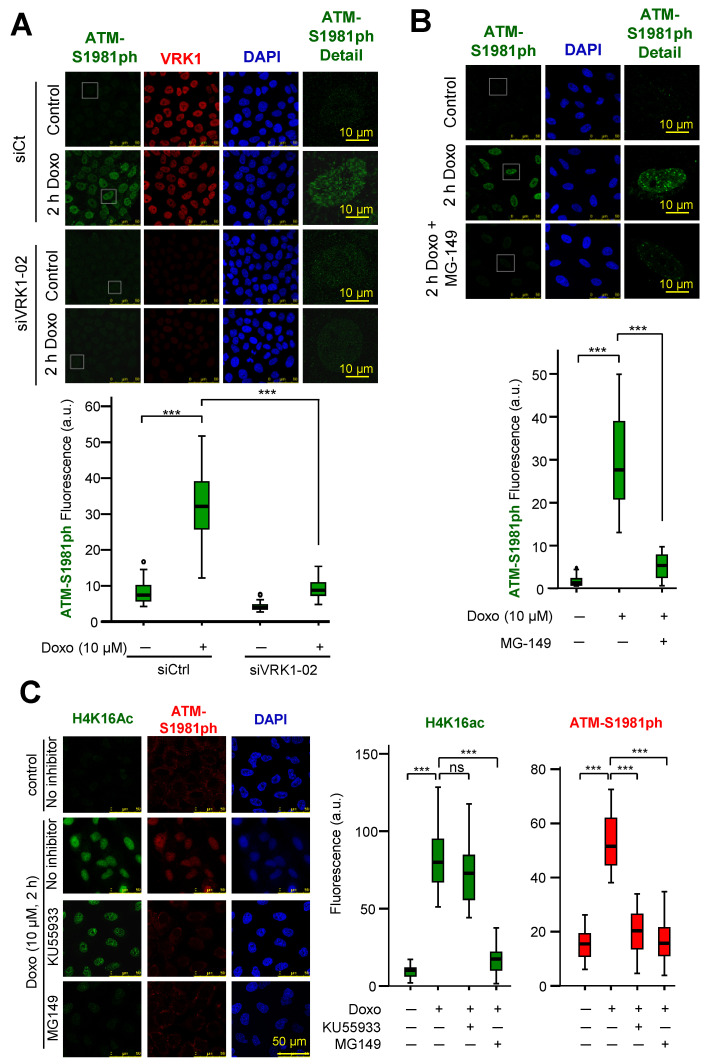
Phosphorylation of ATM is lost by depletion of VRK1 or inhibition of Tip60 in A549 (ATM+/+) cells. (**A**). Effect of VRK1 depletion or Tip60 inhibition on the phosphorylation of ATM in Ser1981. A549 cells deprived of serum (0.5% FBS) for 48 h were treated with doxorubicin and the effect of VRK1 depletion on the phosphorylation of ATM was determined by immunofluorescence. Field images and the selected individual cell are shown in Figure S3A. (**B**). Effect of the inhibition of Tip60 with MG149 on the phosphorylation of ATM in Ser1981 induced by doxorubicin and detected by immunofluorescence. Field images and the indicated individual cell are shown in Figure S3B. (**C**). Differential effect of KU55933 (ATM inhibitor) and MG149 (Tip60 inhibitor) on the acetylation of H4K6 and ATM phosphorylation in A549 cells treated with doxorubicin. The graphs show the quantification of 50 cells. ns: Not significant. *** *p* < 0.001. All experiments were repeated three times.

Next, to confirm this observation at individual cell level, the phosphorylation of ATM induced by doxorubicin and its inhibition, by either VRK1 depletion or the Tip60 inhibitor MG149, were determined by immunofluorescence. VRK1 depletion (Figure 8A) or treatment with MG149 (Figure 8B) resulted in the loss of ATM-S1981 phosphorylation. The KU55933 ATM inhibitor does not affect H4K16 acetylation but prevents ATM-S1981 phosphorylation in the response to doxorubicin (Figure 8C). However, the MG149 Tip60 inhibitor (Figure 8C) prevents both H4K16 acetylation, and ATM-S1981 phosphorylation, which further supports that ATM acetylation precedes its phosphorylation as expected since acetylation of ATM is required for activation of this kinase [56].

### 2.8. The Kinase Activity of VRK1 Is Necessary to Rescue ATM Acetylation and Phosphorylation

**Figure 9:**
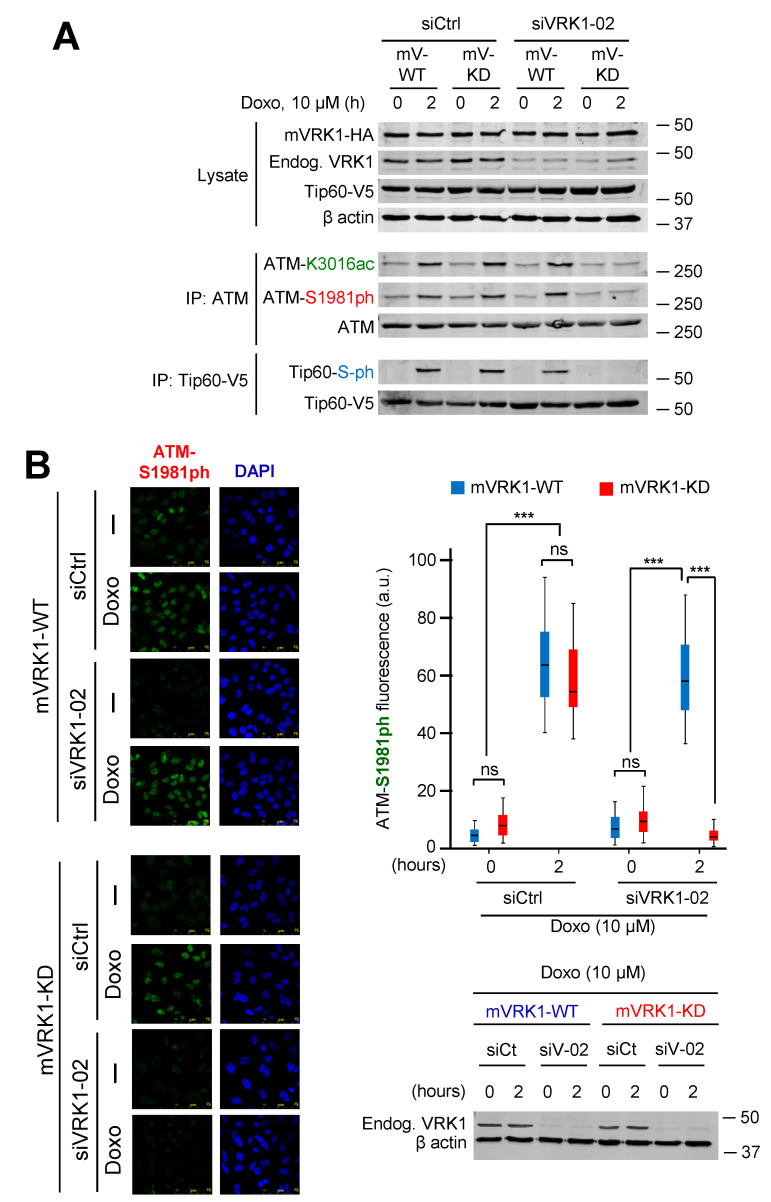
The acetylation and phosphorylation of ATM induced by doxorubicin are rescued by kinase-active VRK1. (**A**). Rescue of ATM acetylation and phosphorylation induced by doxorubicin in A549 stable cell lines expressing kinase-active (mV-WT) (left) or kinase-dead murine VRK1 (K179E) (mV-KD) (right) in which the endogenous human VRK1 was depleted. (**B**). Rescue of ATM phosphorylation induced by doxorubicin in A549 stable cell lines, which express either murine kinase-active (mVRK1) (top) or kinase dead murine VRK1 (K179E) (mVRK1-KD) (bottom), and in which endogenous human VRK1 was depleted. The graph to the right shows the quantification of fifty cells ns: Not significant. *** *p* < 0.001.

To demonstrate that VRK1 is necessary for ATM acetylation and phosphorylation, a different experimental approach was used by performing a rescue experiment. Two A549-derived stable cell lines expressing murine VRK1 kinase active (mVRK1) and kinase-dead (mVRK1-K179E) were used. In these two stable cell lines, the endogenous human VRK1 was depleted and the effect of doxorubicin treatment on endogenous ATM acetylation and phosphorylation were studied, using Tip60 phosphorylation as an internal control. The kinase-active mVRK1 was able to rescue both the specific ATM–K3016 acetylation and ATM-S1981 phosphorylation in immunoblots (Figure 9A), and ATM-S1981 phosphorylation by immunofluorescence (Figure 9B). Neither of these two ATM modifications were rescued by the kinase-dead mVRK1-K179E (Figure 9A,B).

## 3. Discussion

Chromatin remodeling is a highly organized and regulated process. When DNA damage occurs, the cell has to respond to an aberrant alteration by a locally altered chromatin, which triggers a response aiming to its repair and that requires several sequential steps. The many different alternative covalent modifications of histones in chromatin underlie its dynamic changes associated to specific functions. These modifications require several different enzymatic activities that need to be coordinated and linked to specific cellular functions, ranging from transcription, replication, or chromatin compaction to DNA damage responses. There is a large group of lysine acetyl transferases (KAT) [62] and their regulation and coordination are unknown, but in both processes, phosphorylation is likely to play an important role that remains to be studied and understood. Kinases are well suited to orchestrate these roles and associate them to specific signaling pathways. The identification of the chromatin kinase VRK1, which participates in transcription, cell division and DNA repair, as a regulator of KAT5/Tip60 is a significant step in this context. Tip60 phosphorylation occurs in other biological processes. In apoptosis, GSK3 phosphorylates Tip60 and leads to the expression of PUMA (P53 Up-Regulated Modulator of Apoptosis) [63]. Tip60 is also phosphorylated by CDK9 and associated to gene transcription [64], and by cyclinB/cdc2 complexes in G2/M in cell cycle progression [65]. However, the regulation of Tip60 in DNA damage responses is initiated in a locally altered chromatin [48,66], as it occurs with VRK1 that is activated independently of the type of DNA damage [19].

Chromatin kinases are likely candidates to participate in early responses to DNA damage. The location of VRK1 as a kinase resident on chromatin places it in a very suitable situation to trigger, coordinate and organize the signals involved in sequential DDR steps. The colocalization of VRK1 [34] and Tip60 [42,67] on the chromatin fraction and its stable direct interaction indicate that Tip60 phosphorylation can contribute to its retention on this fraction, since VRK1 depletion results in the loss of Tip60 in the chromatin fraction. The complex between VRK1-Tip60 is activated by the structural change caused by the interaction of VRK1 with histones in nucleosomes located where DNA is altered by damage [21,39]. The kinase activity of VRK1 can be regulated by the interaction of its flexible C-terminus with histones [32,39]. These results indicated that the phosphorylation of Tip60 occurred on chromatin where VRK1 is also present and both form a basal complex, but an additional contribution of their nucleoplasm fraction cannot be rule out.

The chromatin kinase VRK1, which indirectly regulates histone acetylation in response to DNA damage in G0/G1, is a likely candidate to regulate Tip60. This KAT also regulates histone acetylation in DDR [45], and VRK1 depletion prevents H4K16 acetylation. The acetylation of H4K16, mediated by Tip60, is a marker of locally relaxed chromatin, facilitates the next steps, after deacetylation, for recruitment of 53BP1 to damaged DNA sites, and DNA repair by the NHEJ pathway [68,69]. Moreover, during progression of DDR, the deacetylation of H4K16 permits the dimethylation of H4K20 (me2) that facilitates later steps in the specific repair pathway [47,69]. Consistent with this role for H4K16ac, depletion of VRK1 impairs later steps in the specific pathway, such as the formation of 53BP1 foci in response to DNA damage by either ionizing radiation or doxorubicin [19,20,21,52], which is not rescued by kinase-dead VRK1 [55]. Thus, the interplay of histone acetylation and methylation can be associated to different functions in DDR, and is consistent with the role of VRK1 in NHEJ in cells in G0/G1. In this context, it will be important to study the role that VRK1 plays in the regulation of the methylase and demethylase that regulate K4H20me2.

**Figure 10:**
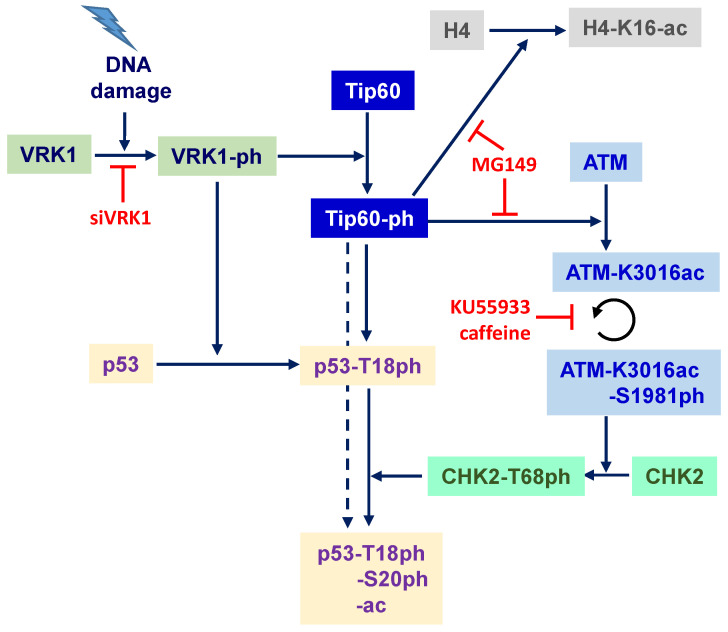
Sequential order of covalent modifications in response to DNA damage involving VRK1, Tip60, H4, ATM and p53. The site of action of siVRK1 and of specific acetylase and kinase inhibitors are indicated.

The sequential order of Tip60 and ATM roles in the early response to DNA damage involving VRK1 is outlined in Figure 10. The interaction of VRK1 with histones regulates its kinase activity and permits the phosphorylation of Tip60, which by acetylation of H4 permits a local chromatin relaxation at the site of DNA damage. This locally relaxed chromatin permits the access to different components that participate in the sequential steps of DNA repair, from the initial DNA-end protection, damage identification and finally repair by facilitating the ATM acetylation and its subsequent autophosphorylation. In the case of DNA damage by doxorubicin that causes double-strand breaks, VRK1 depletion also alters the specific phosphorylation and formation of γH2AX [21], NBS1 [20] and 53BP1 foci [19,52], which represents sequential steps in the process downstream of H4K16ac. Inhibition of ATM, prevents its phosphorylation, but does not alter its acetylation by Tip60, which is lost by VRK1 depletion. Furthermore, there is a parallel cellular response mediated by VRK1 in response to DNA damage involving the specific phosphorylation of p53 in Thr18 [24], which prevents its interaction with mdm2, and facilitates subsequent phosphorylation by ATM/ATR to select specific transcriptional cofactors [70]. The initial phosphorylation of p53 in Thr18 by VRK1 [23,24,71] disrupting the interaction between p53 and the mdm2 ubiquitin ligase [72]. Additional phosphorylation of p53 in other N-terminus residues, such as Ser15 by ATR and Ser20 by ATM [73], determine the selection and affinity of p53 for specific transcriptional cofactors [70]. A similar order is likely to occur in the coordination of sequential steps in DDR. The sequential order of activation of kinases in DDR is consistent with their consecutive roles in the phosphorylation of p53 in response to DNA damage. This p53 activation by VRK1 contributes to induction of cell cycle arrest or apoptosis, depending on the magnitude of the DNA damage, and the ability of the cell to cope with it.

In this study, we have identified that VRK1 regulates the phosphorylation of Tip60 in the absence of ATM and in the presence of ATM inhibitors, indicating that VRK1 is an upstream component needed for the H4K16ac associated to the aberrant relaxation of damaged DNA and required for initiation of the specific sequential steps in DDR. This finding, the activation of Tip60/KAT5, a writer epigenetic enzyme, is a step in the coordination and regulation of alternative modifications in specific histone lysine residues. In those lysine residues with alternative epigenetic modifications, the coordination of four enzymes involved is still poorly known, but that is essential to understand how chromatin perform its transition from one configuration to another and to organize its three-dimensional structure in different physiological or pathological situations.

## 4. Materials and Methods

### 4.1. Reagents

MG149 (Axon MedChem, Groningen, The Netherlands), C646 (SelleckChem, Houston, TX, USA), KU55933 (Tocris Bioscience, Bristol, UK) were dissolved in dimethyl sulfoxide. All other reagents were from Sigma-Aldrich-Merck (Darmstadt, Germany).

### 4.2. Plasmids

Human VRK1 was expressed from plasmid pCEFL-HA-VRK1 [18,20,74]. Human Tip60 was expressed from plasmid pcDNA3.1-TIP60-V5-His obtained from D. Maurer [63]. Murine VRK1 (mVRK1) was expressed from plasmid pCMV6-mVRK1-myc-DKK (OriGene, Rockville, MD, USA). A kinase-dead construct of the murine VRK1(K179E) was generated with the Quick-Mutagenesis system (Stratagene, San Diego, CA, USA) [74]. Murine VRK1, wild-type and kinase dead (K179E) were also made in lentiviral construct, plasmids pLenti-C-HA-IRES-BSD-mVRK1 and pLenty-C-HA-IRES-BSD-mVRK(K179E) were used to make A549-derived stable cell lines that were selected with blasticidine.

For in vitro kinase assays of the human protein, the following plasmids were used, PGEX-4T-VRK1 and pGEX-4T-VRK1-K179E [24,55,75] for expression and purification of the fusion proteins expressed in *Escherichia coli* DH5α and the GST-fusion proteins were purified as previously reported [26,75]. Human Tip60 cloned in vector pET28a-LIC (HTATIP-2OU2) with a hexahistidine tag for bacterial expression was a gift from Cheryl Arrowsmith (Addgene reference 33338). Mutations introduced in human of murine were confirmed by Sanger sequencing and reported in previous studies [19,20].

### 4.3. Cell Lines, Culture and Transfections

The following cell lines were used and obtained from the ATCC (Teddington, Middlesex, UK) A549 (CCL-185) and HT144 (*ATM*−/−) (HTB-63). Cell lines were mycoplasma free. Cells were grown in 100 mm dishes with DMEM medium (GIBCO) supplemented with 10% inactivated fetal bovine serum (GIBCO), 1% L-Glutamine (GIBCO) and 0.5% Pen Strep (GIBCO). Twenty-four hours later, cells were transfected with the corresponding expression plasmid. Transfections were performed mixing the amount of plasmid with two volumes of polyethylenimine (PEI) reagent (Polysciences, Inc, Warrington, PA, USA) following manufacturer’s instructions in 1 mL of 150 mM NaCl, incubated for 30 min at room temperature, and added to the cell culture [18,74]. Cell lysis was performed with mild lysis buffer (50 mM Tris–HCl (pH 8.0), 1 mM EDTA, 150 mM NaCl, and 1% triton X-100). At the time of the lysis, the buffer was complemented with phosphatases inhibitors (1 mM NaF and 1 mM sodium orthovanadate) and proteases inhibitors (1 mM PMFS, 10 µg/mL aprotinin and 10 µg/mL (leupeptin).

In all experiments involving induction of DNA damage by doxorubicin treatment, cells were placed in 0.5% FBS for 48 h before doxorubicin addition to the culture. Serum deprivation was used to remove mitogenic signals from growth factors, and to accumulate cell in G0/G1, which was checked by flow cytometry and the lack of cyclin D1and phosho-Rb [17,19].

A549 cells lines were infected with lentiviral particles containing the vector pLentiC-HA-IRES-BSD (OriGene Technologies, Rockville, MD, USA) expressing murine VRK1 or kinase-dead murine VRK1 (K179E) were cloned and selected with blasticidine [76].

In all time course experiments, the reference value is the one corresponding to the starting time point (0 min), and the sequential time points were compared with it after normalization by the levels of endogenous and transfected protein, where applicable. The inhibitors MG149 (2 µM) or C646 (10 µM) were added 10 h before addition of doxorubicin. All experiments were repeated three times.

### 4.4. VRK1 Depletion by siRNA

Specific silencing of VRK1 was performed using two different siRNA from Dharmacon (DHARMACON RNA Technologies). The VRK1 sequences targeted by these siRNA oligonucleotides were siVRK1-02 (siV1-02), CAAGGAACCTGGTGTTGAA; and siVRK1-03 (siV1-03): That GGAAUGGAAAGUAGGAUUA. As negative control, the “ON-TARGETplus siCONTROL Non-targeting siRNA” from DHARMACON was used and is indicated as siCt in experiments. The efficiency of RNAi transfection was determined with “siGLO RISC-free siRNA” (DHARMACON). Briefly, cells were transfected with the indicated siRNA at a concentration of 20 nM using Lipofectamine 2000 Reagent (Invitrogen) or Lipotransfectin (Nivorlab) [18,34,74]. After transfection, cells were processed at the times indicated in specific experiments that were performed as previously reported [19,77]. The depletion of VRK1 by siRNA has been previously reported for A549 [18,21,36] and HT144 [20,21] cell lines.

### 4.5. Antibodies

The primary and secondary antibodies, applications and conditions used in this work are listed in Tables S1 and S2 respectively.

### 4.6. Immunoblots

Protein extracts from cell lysates were quantified using the Protein assay (Bio-Rad; Hercules, CA, USA). Forty micrograms of protein extracts were used for immunoblots. Protein samples were fractionated in a 7.5%, 10%, or 12.5% SDS polyacrylamide gel and transferred to a PVDF Immobilon-FL membrane (Millipore), at 90 V, 4 °C, for 90 min. PVDF membranes were blocked for 1 h at room temperature with 5% defatted milk in TBS-T buffer (25 mM Tris-HCl pH 8.50 mM NaCl, 2.5 mM KCl, 0.1% Tween-20) or, alternatively, with 5% of BSA in TBS-T buffer when phosphorylated state of proteins was analyzed [20,34,36]. Membranes were washed in TBS-T buffer 3 times for 10 min each time and incubated with the primary antibody for 8 h, or overnight, at 4 °C. Next, PVDF membranes were extensively washed in TBS-T buffer and incubated with the corresponding secondary antibody (Goat Anti-Mouse IgG, Dylight 680 -red colored- or Goat Anti-Rabbit IgG, DyLight 800 -green colored-) at 1:10,000 for 1 h at room temperature (in darkness). Membranes were washed in TBS-T buffer for 10 min three times. Finally, fluorescence signals were detected, and quantified, using a LI-COR Odyssey Infrared Imaging System (LI-COR Biosciences, Lincoln, NE, USA).

### 4.7. Immunoprecipitations

Cells were lysed at a density of seventy percent confluence. Cell lysates (1–2 mg of protein) were incubated with the corresponding antibody for each experiment for 8 h, or overnight, at 4 °C with agitation. Subsequently, the protein-antibody immune complexes were precipitated with 80 µL of Protein G–Agarose Resin 4 Rapid Run (4RRPG, Agarose Bead Technologies) for 8 h at 4 °C in agitation [18,34,74] and the immunoprecipitate was collected by centrifugation (2200 rpm, 2 min) and washed three times with lysis buffer. Next, 10 µL of buffer 5× (250 mM Tris-HCl, 10% SDS, 50% glycerol, 0.5% bromophenol blue) were added to the samples, boiled for 5 min and subjected to electrophoresis, followed by immunoblot analysis. Specific antibodies for immunoblot analysis are listed in Table S1.

### 4.8. In Vitro VRK1-Tip60 Protein Interaction and Kinase Assay

The kinase assay contained, either 10 µg of purified GST-VRK1 wild-type or 10 µg of purified GST-VRK1-K179E (kinase-dead) with 6 µg of purified His-Tip60 as substrate, to maintain an equimolar ratio. The protein mix was incubated in a specific buffer for casein kinases (20 mM Tris-HCl pH 7.5, 5 mM MgCl_2_, 0.5 mM DTT and 150 mM KCl) and 2 mM of cold ATP during 1 h at 30 °C. The proteins fractionated in SDS-polyacrylamide gels, and transferred to an Inmobilon-FL membrane (Millipore). Tip60 phosphorylation was detected with monoclonal anti-phosphoserine antibody (clone 4A4). The proteins on the membrane were detected by Western blot.

To study the interaction between human VRK1 and Tip60 a binding assay was performed using bacterially expressed and purified GST-VRK1 and His-Tip60 in the amounts indicated in the experiment [74,75]. The proteins were incubated in a buffer containing 20 mM Tris-HCl pH 7.5, 5 mM MgCl_2_, 0.5 mM DTT and 150 mM KCl in a volume of 25 µL at 35 °C and gentle agitation for 1 h, followed by the addition of 80 µL of Gutathione-sepharose 4 Fast Flow (Merck, ref: GE17-5132-01) equilibrated with lysis buffer. The mix was incubated at 4 °C for eight hours. Finally, a pull-down was performed by centrifugation at 2800 rpm for 5 min at 4 °C. The resin was washed three times in the same pull-down buffer, and finally 10 µL of buffer were added and loaded in a 10% polyacrylamide-SDS gel. The proteins were transferred to a PVDF Immobilon-FL membrane that were used for immunoblot analysis.

### 4.9. Immunofluorescence and Confocal Microscopy

Cells were cultured in coverslips placed in 60-mm dishes. Forty-eight or ninety-six hours after corresponding transfections of expression plasmids and/or siRNAs, coverslips were placed into T24 single wells and cells were fixed with 3% paraformaldehyde (MERCK) in 1× PBS during 30 min at room temperature. Next, cells were treated with 200 mM glycine during 15 min and permeabilized with 0.2% Triton X-100 in PBS for 30 min. Finally, cells were blocked with 1% BSA in PBS 1× for, at least, 1 h. Once permeabilized, coverslips were incubated with the corresponding first primary antibody in 1% BSA in PBS for 8 h, or overnight, at 4 °C. Afterwards, cells were washed 3 times with PBS, and the secondary antibody was added and incubated and washed in a similar manner. The specific antibodies and dilutions used for this technique are indicated in Table S1. The secondary antibodies (Table S2) were incubated together in 1% BSA in PBS for 1 h at room temperature. Finally, cell nuclei were stained with 0.1% DAPI (SIGMA) in 1× PBS for 15 min at room temperature and washed 3 times with PBS. Coverslips were mounted with Mowiol (Calbiochem-Merck), and images were captured with a LEICA SP5 DMI-6000B confocal microscope. The lasers used in this microscope were: Argon (488 nm) for detecting green fluorescence, DPSS (561 nm) for detecting red fluorescence, and UV Diode (405 nm) for DAPI detection. Images were captured with a 63.0× lens zoomed in 2× with a 1024 × 1024 frame and 600 Hz scanning speed. Images were analyzed with the ImageJ program (NIH).

### 4.10. Nucleoplasm-Chromatin Fractionation

Fractionation of nucleoplasm and chromatin fraction was performed as reported for Tip60 [64]. Briefly, cells were lysed and incubated on ice for 8 min in cytosol lysis buffer (10 mM HEPES; 10 mM KCl, 1.5 mM MgCl_2_, 0.34 mM sucrose, 10% glycerol, 20 µM MG132, 0.1% Triton X-100, 1:100 PMSF, 1:1000 aprotinin, 1:1000 leupeptin, 1:500 sodium orthovanadate and 1:500 NaF). Subsequently, cell lysate was centrifuged at 1300× *g* for 5 min in order to separate cytoplasm fraction (supernatant), which was discarded, and whole nuclei extract (pellet). Nuclei fraction was taken up in nucleoplasm extracting buffer (400 mM NaCl, 3 mM EDTA, 0.2 mM EGTA, 1 mM DTT, 20 µM MG132, 1:100 PMSF, 1:1000 aprotinin, 1:1000 leupeptin, 1:500 sodium orthovanadate and 1:500 NaF) and incubated at 4 °C for 20 min. To precipitate chromatin from nucleoplasm (supernatant) an additional centrifugation at 1000× *g* for 2 min was performed. The chromatin fraction was washed three times in nucleoplasm extracting buffer. After that, the chromatin fraction was resuspended in nuclei lysis buffer (50 mM Tris-HCl, pH 8.1, 10 mM EDTA, 0.5% SDS, 1:100 PMSF, 1:1000 aprotinin, 1:1000 leupeptin, 1:500 sodium orthovanadate and 1:500 NaF) and sonicated 5 times for 30 seconds, waiting 30 seconds between each sonication to break DNA. Nucleoplasm and chromatin-associated proteins were quantified by Bradford assay, and analyzed by Western blotting.

### 4.11. Statistical Analysis

Statistical results were analyzed using the SPSS23 statistical package. Quantitative experiments were performed at least three times, the number of cells is indicated and statistical analysis was performed by the non-parametric Mann–Whitney U test after confirming samples did not adjust to a normal distribution according to two-tailed Kolmogorov test using the IBM SPSS 23 statistics package. Results are presented as box plots with the median, first and third quartiles and standard deviations [78].

### 4.12. TUNEL Assay

TUNEL (TdT-mediated dUTP Nick-End Labeling) was performed to analyze the DNA free ends of DNA breaks associated to doxorubicin treatments following the indications of the provider (In Situ Cell Death Detection Kit, Fluorescein; Ref: 11684795910; Roche). Cells were cultured in coverslips placed in 60-mm dishes under the specific conditions of the experiments and, subsequently, they were fixed and permeabilized as indicated for immunofluorescence assays. Simultaneously, 50 µL of enzyme solution, containing terminal deoxynucleotidyl transferase (TdT), were mixed with 550 µL of label solution, which contains nucleotide mixture in reaction buffer. The coverslips were incubated with 50 µL of the TUNEL reaction mixture in a humidified atmosphere for 60 min at 37 °C in the dark. Later, coverslips were rinse 3 times with PBS and samples were analyzed in the confocal microscope using an excitation wavelength of 488 nm and detection in the range of 515–565 nm (green).

### 4.13. Database Submission

The protein interactions from this publication have been submitted to the IMEx (http://www.imexconsortium.org) consortium through IntAct [79], and assigned the identifier IM-27878.

## 5. Conclusions

We have identified that VRK1 regulates the phosphorylation of Tip60 in the absence of ATM and in the presence of ATM inhibitors, indicating that VRK1 is an upstream component needed for the H4K16ac associated to the aberrant relaxation of damaged DNA and required for initiation of the specific sequential steps in DDR.

## Supplementary Materials

The following are available online at https://www.mdpi.com/2072-6694/12/10/2986/s1, Figure S1. Induction of DNA damage by doxorubicin and triggering a DNA-Damage response (DDR) by the NHEJ pathway in A549 cells. Figure S2. Effect of acetyl transferase inhibitors on the basal level of endogenous H4K16 acetylation. Figure S3. Phosphorylation of ATM is lost by either depletion of VRK1 or inhibition of Tip60 in A549 (ATM+/+) cells. Field images. Figure S4. Uncropped Western Blotting figures. Table S1. Primary antibodies, conditions of applications. Table S2. Secondary antibodies, conditions of applications.

## References

[1] Smith E., Shilatifard A. (2010). The chromatin signaling pathway: Diverse mechanisms of recruitment of histone-modifying enzymes and varied biological outcomes. Mol. Cell.

[2] Bannister A.J., Kouzarides T. (2011). Regulation of chromatin by histone modifications. Cell Res..

[3] Dawson M.A., Kouzarides T. (2012). Cancer epigenetics: From mechanism to therapy. Cell.

[4] Tessarz P., Kouzarides T. (2014). Histone core modifications regulating nucleosome structure and dynamics. Nat. Rev. Mol. Cell. Biol..

[5] Ting A.H., McGarvey K.M., Baylin S.B. (2006). The cancer epigenome–components and functional correlates. Genes Dev..

[6] Beekman R., Chapaprieta V., Russinol N., Vilarrasa-Blasi R., Verdaguer-Dot N., Martens J.H.A., Duran-Ferrer M., Kulis M., Serra F., Javierre B.M. (2018). The reference epigenome and regulatory chromatin landscape of chronic lymphocytic leukemia. Nat. Med..

[7] Mazor T., Pankov A., Song J.S., Costello J.F. (2016). Intratumoral Heterogeneity of the Epigenome. Cancer Cell.

[8] Wysocka J., Swigut T., Xiao H., Milne T.A., Kwon S.Y., Landry J., Kauer M., Tackett A.J., Chait B.T., Badenhorst P. (2006). A PHD finger of NURF couples histone H3 lysine 4 trimethylation with chromatin remodelling. Nature.

[9] Xhemalce B., Kouzarides T. (2010). A chromodomain switch mediated by histone H3 Lys 4 acetylation regulates heterochromatin assembly. Genes Dev..

[10] Murata K., Kouzarides T., Bannister A.J., Gurdon J.B. (2010). Histone H3 lysine 4 methylation is associated with the transcriptional reprogramming efficiency of somatic nuclei by oocytes. Epigenet. Chromatin.

[11] Kouzarides T. (2007). Chromatin modifications and their function. Cell.

[12] Dawson M.A., Kouzarides T., Huntly B.J. (2012). Targeting epigenetic readers in cancer. N. Engl. J. Med..

[13] Cantarero L., Moura D.S., Salzano M., Monsalve D.M., Campillo-Marcos I., Martín-Doncel E., Lazo P.A., Choi S. (2017). VRK1 (vaccinia-related kinase 1). Encyclopedia of Signaling Molecules.

[14] Aihara H., Nakagawa T., Mizusaki H., Yoneda M., Kato M., Doiguchi M., Imamura Y., Higashi M., Ikura T., Hayashi T. (2016). Histone H2A T120 Phosphorylation Promotes Oncogenic Transformation via Upregulation of Cyclin D1. Mol. Cell.

[15] Varjosalo M., Sacco R., Stukalov A., van Drogen A., Planyavsky M., Hauri S., Aebersold R., Bennett K.L., Colinge J., Gstaiger M. (2013). Interlaboratory reproducibility of large-scale human protein-complex analysis by standardized AP-MS. Nat. Methods.

[16] Santos C.R., Rodriguez-Pinilla M., Vega F.M., Rodriguez-Peralto J.L., Blanco S., Sevilla A., Valbuena A., Hernandez T., van Wijnen A.J., Li F. (2006). VRK1 signaling pathway in the context of the proliferation phenotype in head and neck squamous cell carcinoma. Mol. Cancer Res..

[17] Valbuena A., Lopez-Sanchez I., Lazo P.A. (2008). Human VRK1 is an early response gene and its loss causes a block in cell cycle progression. PLoS ONE.

[18] Moura D.S., Fernández I.F., Marín-Royo G., López-Sánchez I., Martín-Doncel E., Vega F.M., Lazo P.A. (2016). Oncogenic Sox2 regulates and cooperates with VRK1 in cell cycle progression and differentiation. Sci. Rep..

[19] Sanz-Garcia M., Monsalve D.M., Sevilla A., Lazo P.A. (2012). Vaccinia-related Kinase 1 (VRK1) is an upstream nucleosomal kinase required for the assembly of 53BP1 foci in response to ionizing radiation-induced DNA damage. J. Biol. Chem..

[20] Monsalve D.M., Campillo-Marcos I., Salzano M., Sanz-Garcia M., Cantarero L., Lazo P.A. (2016). VRK1 phosphorylates and protects NBS1 from ubiquitination and proteasomal degradation in response to DNA damage. Biochim. Biophys. Acta Mol. Cell Res..

[21] Salzano M., Sanz-Garcia M., Monsalve D.M., Moura D.S., Lazo P.A. (2015). VRK1 chromatin kinase phosphorylates H2AX and is required for foci formation induced by DNA damage. Epigenetics.

[22] Campillo-Marcos I., Lazo P.A. (2018). Implication of the VRK1 chromatin kinase in the signaling responses to DNA damage: A therapeutic target?. Cell Mol. Life Sci..

[23] Lopez-Borges S., Lazo P.A. (2000). The human vaccinia-related kinase 1 (VRK1) phosphorylates threonine-18 within the mdm-2 binding site of the p53 tumour suppressor protein. Oncogene.

[24] Vega F.M., Sevilla A., Lazo P.A. (2004). p53 Stabilization and accumulation induced by human vaccinia-related kinase 1. Mol. Cell. Biol..

[25] Valbuena A., Vega F.M., Blanco S., Lazo P.A. (2006). p53 downregulates its activating vaccinia-related kinase 1, forming a new autoregulatory loop. Mol. Cell. Biol..

[26] Sevilla A., Santos C.R., Barcia R., Vega F.M., Lazo P.A. (2004). c-Jun phosphorylation by the human vaccinia-related kinase 1 (VRK1) and its cooperation with the N-terminal kinase of c-Jun (JNK). Oncogene.

[27] Sevilla A., Santos C.R., Vega F.M., Lazo P.A. (2004). Human vaccinia-related kinase 1 (VRK1) activates the ATF2 transcriptional activity by novel phosphorylation on Thr-73 and Ser-62 and cooperates with JNK. J. Biol. Chem..

[28] Kang T.H., Park D.Y., Kim W., Kim K.T. (2008). VRK1 phosphorylates CREB and mediates CCND1 expression. J. Cell. Sci..

[29] Jamin A., Wiebe M.S. (2015). Barrier to Autointegration Factor (BANF1): Interwoven roles in nuclear structure, genome integrity, innate immunity, stress responses and progeria. Curr. Opin. Cell Biol..

[30] Wiebe M.S., Jamin A. (2016). The Barrier to Autointegration Factor: Interlocking Antiviral Defense with Genome Maintenance. J. Virol..

[31] Samwer M., Schneider M.W.G., Hoefler R., Schmalhorst P.S., Jude J.G., Zuber J., Gerlich D.W. (2017). DNA Cross-Bridging Shapes a Single Nucleus from a Set of Mitotic Chromosomes. Cell.

[32] Kang T.H., Park D.Y., Choi Y.H., Kim K.J., Yoon H.S., Kim K.T. (2007). Mitotic histone H3 phosphorylation by vaccinia-related kinase 1 in mammalian cells. Mol. Cell. Biol..

[33] Vazquez-Cedeira M., Barcia-Sanjurjo I., Sanz-Garcia M., Barcia R., Lazo P.A. (2011). Differential Inhibitor Sensitivity between Human Kinases VRK1 and VRK2. PLoS ONE.

[34] Moura D.S., Campillo-Marcos I., Vazquez-Cedeira M., Lazo P.A. (2018). VRK1 and AURKB form a complex that cross inhibit their kinase activity and the phosphorylation of histone H3 in the progression of mitosis. Cell. Mol. Life Sci..

[35] Aihara H., Nakagawa T., Yasui K., Ohta T., Hirose S., Dhomae N., Takio K., Kaneko M., Takeshima Y., Muramatsu M. (2004). Nucleosomal histone kinase-1 phosphorylates H2A Thr 119 during mitosis in the early Drosophila embryo. Genes Dev..

[36] Campillo-Marcos I., Lazo P.A. (2019). Olaparib and ionizing radiation trigger a cooperative DNA-damage repair response that is impaired by depletion of the VRK1 chromatin kinase. J. Exp. Clin. Cancer Res..

[37] Choi Y.H., Lim J.K., Jeong M.W., Kim K.T. (2012). HnRNP A1 phosphorylated by VRK1 stimulates telomerase and its binding to telomeric DNA sequence. Nucleic Acids Res..

[38] Shin J., Chakraborty G., Bharatham N., Kang C., Tochio N., Koshiba S., Kigawa T., Kim W., Kim K.T., Yoon H.S. (2011). NMR solution structure of human vaccinia-related kinase 1 (VRK1) reveals the C-terminal tail essential for its structural stability and autocatalytic activity. J. Biol. Chem..

[39] Kim W., Chakraborty G., Kim S., Shin J., Park C.H., Jeong M.W., Bharatham N., Yoon H.S., Kim K.T. (2012). Macro Histone H2A1.2 (MacroH2A1) Protein Suppresses Mitotic Kinase VRK1 during Interphase. J. Biol. Chem..

[40] Sanz-Garcia M., Lopez-Sanchez I., Lazo P.A. (2008). Proteomics identification of nuclear Ran GTPase as an inhibitor of human VRK1 and VRK2 (vaccinia-related kinase) activities. Mol. Cell. Proteom..

[41] Robinson P.J., An W., Routh A., Martino F., Chapman L., Roeder R.G., Rhodes D. (2008). 30 nm chromatin fibre decompaction requires both H4-K16 acetylation and linker histone eviction. J. Mol. Biol..

[42] Murr R., Loizou J.I., Yang Y.G., Cuenin C., Li H., Wang Z.Q., Herceg Z. (2006). Histone acetylation by Trrap-Tip60 modulates loading of repair proteins and repair of DNA double-strand breaks. Nat. Cell. Biol..

[43] Stante M., Minopoli G., Passaro F., Raia M., Vecchio L.D., Russo T. (2009). Fe65 is required for Tip60-directed histone H4 acetylation at DNA strand breaks. Proc. Natl. Acad. Sci. USA.

[44] Krishnan V., Chow M.Z., Wang Z., Zhang L., Liu B., Liu X., Zhou Z. (2011). Histone H4 lysine 16 hypoacetylation is associated with defective DNA repair and premature senescence in Zmpste24-deficient mice. Proc. Natl. Acad. Sci. USA.

[45] Ikura M., Furuya K., Matsuda S., Matsuda R., Shima H., Adachi J., Matsuda T., Shiraki T., Ikura T. (2015). Acetylation of Histone H2AX at Lys 5 by the TIP60 Histone Acetyltransferase Complex Is Essential for the Dynamic Binding of NBS1 to Damaged Chromatin. Mol. Cell. Biol..

[46] Jacquet K., Fradet-Turcotte A., Avvakumov N., Lambert J.P., Roques C., Pandita R.K., Paquet E., Herst P., Gingras A.C., Pandita T.K. (2016). The TIP60 Complex Regulates Bivalent Chromatin Recognition by 53BP1 through Direct H4K20me Binding and H2AK15 Acetylation. Mol. Cell.

[47] Renaud E., Barascu A., Rosselli F. (2016). Impaired TIP60-mediated H4K16 acetylation accounts for the aberrant chromatin accumulation of 53BP1 and RAP80 in Fanconi anemia pathway-deficient cells. Nucleic Acids Res..

[48] Sun Y., Jiang X., Chen S., Fernandes N., Price B.D. (2005). A role for the Tip60 histone acetyltransferase in the acetylation and activation of ATM. Proc. Natl. Acad. Sci. USA.

[49] Chen G., Cheng Y., Tang Y., Martinka M., Li G. (2012). Role of Tip60 in human melanoma cell migration, metastasis, and patient survival. J. Investig. Dermatol..

[50] Miyamoto N., Izumi H., Noguchi T., Nakajima Y., Ohmiya Y., Shiota M., Kidani A., Tawara A., Kohno K. (2008). Tip60 is regulated by circadian transcription factor clock and is involved in cisplatin resistance. J. Biol. Chem..

[51] Finetti P., Cervera N., Charafe-Jauffret E., Chabannon C., Charpin C., Chaffanet M., Jacquemier J., Viens P., Birnbaum D., Bertucci F. (2008). Sixteen-kinase gene expression identifies luminal breast cancers with poor prognosis. Cancer Res..

[52] Salzano M., Vazquez-Cedeira M., Sanz-Garcia M., Valbuena A., Blanco S., Fernandez I.F., Lazo P.A. (2014). Vaccinia-related kinase 1 (VRK1) confers resistance to DNA-damaging agents in human breast cancer by affecting DNA damage response. Oncotarget.

[53] Li L., Wang Y. (2017). Cross-talk between the H3K36me3 and H4K16ac histone epigenetic marks in DNA double-strand break repair. J. Biol. Chem..

[54] Bowers E.M., Yan G., Mukherjee C., Orry A., Wang L., Holbert M.A., Crump N.T., Hazzalin C.A., Liszczak G., Yuan H. (2010). Virtual ligand screening of the p300/CBP histone acetyltransferase: Identification of a selective small molecule inhibitor. Chem. Biol..

[55] Martin-Doncel E., Rojas A.M., Cantarero L., Lazo P.A. (2019). VRK1 functional insufficiency due to alterations in protein stability or kinase activity of human VRK1 pathogenic variants implicated in neuromotor syndromes. Sci. Rep..

[56] Sun Y., Xu Y., Roy K., Price B.D. (2007). DNA damage-induced acetylation of lysine 3016 of ATM activates ATM kinase activity. Mol. Cell. Biol..

[57] Kozlov S.V., Graham M.E., Jakob B., Tobias F., Kijas A.W., Tanuji M., Chen P., Robinson P.J., Taucher-Scholz G., Suzuki K. (2011). Autophosphorylation and ATM activation: Additional sites add to the complexity. J. Biol. Chem..

[58] Zhang T., Shen Y., Chen Y., Hsieh J.T., Kong Z. (2015). The ATM inhibitor KU55933 sensitizes radioresistant bladder cancer cells with DAB2IP gene defect. Int. J. Radiat. Biol..

[59] Carruthers R., Ahmed S.U., Strathdee K., Gomez-Roman N., Amoah-Buahin E., Watts C., Chalmers A.J. (2015). Abrogation of radioresistance in glioblastoma stem-like cells by inhibition of ATM kinase. Mol. Oncol..

[60] Sarkaria J.N., Busby E.C., Tibbetts R.S., Roos P., Taya Y., Karnitz L.M., Abraham R.T. (1999). Inhibition of ATM and ATR kinase activities by the radiosensitizing agent, caffeine. Cancer Res..

[61] Zhou B.B., Chaturvedi P., Spring K., Scott S.P., Johanson R.A., Mishra R., Mattern M.R., Winkler J.D., Khanna K.K. (2000). Caffeine abolishes the mammalian G(2)/M DNA damage checkpoint by inhibiting ataxia-telangiectasia-mutated kinase activity. J. Biol. Chem..

[62] Roos W.P., Krumm A. (2016). The multifaceted influence of histone deacetylases on DNA damage signalling and DNA repair. Nucleic Acids Res..

[63] Charvet C., Wissler M., Brauns-Schubert P., Wang S.J., Tang Y., Sigloch F.C., Mellert H., Brandenburg M., Lindner S.E., Breit B. (2011). Phosphorylation of Tip60 by GSK-3 determines the induction of PUMA and apoptosis by p53. Mol. Cell.

[64] Brauns-Schubert P., Schubert F., Wissler M., Weiss M., Schlicher L., Bessler S., Safavi M., Miething C., Borner C., Brummer T. (2018). CDK9-mediated phosphorylation controls the interaction of TIP60 with the transcriptional machinery. EMBO Rep..

[65] Lemercier C., Legube G., Caron C., Louwagie M., Garin J., Trouche D., Khochbin S. (2003). Tip60 acetyltransferase activity is controlled by phosphorylation. J. Biol. Chem..

[66] Sun Y., Jiang X., Price B.D. (2010). Tip60: Connecting chromatin to DNA damage signaling. Cell Cycle.

[67] Chailleux C., Tyteca S., Papin C., Boudsocq F., Puget N., Courilleau C., Grigoriev M., Canitrot Y., Trouche D. (2010). Physical interaction between the histone acetyl transferase Tip60 and the DNA double-strand breaks sensor MRN complex. Biochem. J..

[68] Hsiao K.Y., Mizzen C.A. (2013). Histone H4 deacetylation facilitates 53BP1 DNA damage signaling and double-strand break repair. J. Mol. Cell. Biol..

[69] Tang J., Cho N.W., Cui G., Manion E.M., Shanbhag N.M., Botuyan M.V., Mer G., Greenberg R.A. (2013). Acetylation limits 53BP1 association with damaged chromatin to promote homologous recombination. Nat. Struct. Mol. Biol..

[70] Teufel D.P., Bycroft M., Fersht A.R. (2009). Regulation by phosphorylation of the relative affinities of the N-terminal transactivation domains of p53 for p300 domains and Mdm2. Oncogene.

[71] Lopez-Sanchez I., Valbuena A., Vazquez-Cedeira M., Khadake J., Sanz-Garcia M., Carrillo-Jimenez A., Lazo P.A. (2014). VRK1 interacts with p53 forming a basal complex that is activated by UV-induced DNA damage. FEBS Lett..

[72] Kussie P.H., Gorina S., Marechal V., Elenbaas B., Moreau J., Levine A.J., Pavletich N.P. (1996). Structure of the MDM2 oncoprotein bound to the p53 tumor suppressor transactivation domain. Science.

[73] Toledo F., Wahl G.M. (2006). Regulating the p53 pathway: In vitro hypotheses, in vivo veritas. Nat. Rev. Cancer.

[74] Cantarero L., Sanz-Garcia M., Vinograd-Byk H., Renbaum P., Levy-Lahad E., Lazo P.A. (2015). VRK1 regulates Cajal body dynamics and protects coilin from proteasomal degradation in cell cycle. Sci. Rep..

[75] Lopez-Sanchez I., Sanz-Garcia M., Lazo P.A. (2009). Plk3 interacts with and specifically phosphorylates VRK1 in Ser342, a downstream target in a pathway that induces Golgi fragmentation. Mol. Cell. Biol..

[76] Marcos A.T., Martin-Doncel E., Morejon-Garcia P., Marcos-Alcalde I., Gomez-Puertas P., Segura-Puimedon M., Armengol L., Navarro-Pando J.M., Lazo P.A. (2020). VRK1 (Y213H) homozygous mutant impairs Cajal bodies in a hereditary case of distal motor neuropathy. Ann. Clin. Transl. Neurol..

[77] Valbuena A., Castro-Obregon S., Lazo P.A. (2011). Downregulation of VRK1 by p53 in Response to DNA Damage Is Mediated by the Autophagic Pathway. PLoS ONE.

[78] Bremer M., Doerge R.M. (2009). Statistics at the Bench: A Step-By Step Handbook for Biologists.

[79] Orchard S., Ammari M., Aranda B., Breuza L., Briganti L., Broackes-Carter F., Campbell N.H., Chavali G., Chen C., del-Toro N. (2014). The MIntAct project--IntAct as a common curation platform for 11 molecular interaction databases. Nucleic Acids Res..

