# Supplementary material for: VRK1 Phosphorylates Tip60/KAT5 and Is Required for H4K16 Acetylation in Response to DNA Damage
Source: Cancers (Basel). 2020 Oct 15;12(10):2986. doi: 10.3390/cancers12102986 (PMC7650776; doi:10.3390/cancers12102986)

Supplementary Materials

VRK1 Phosphorylates Tip60/KAT5 and is Required for H4K16 Acetylation in Response to DNA Damage

Raúl García-González, Patricia Morejón-García, Ignacio Campillo-Marcos, Marcella Salzano and Pedro A. Lazo

**Figure S1A.** Induction of DNA damage by doxorubicin and triggering a DNA-Damage response (DDR) by the NHEJ pathway in A549 cells. Doxorubicin induces DNA damage that is detected by labeling free DNA ends with TdT (Terminal deoxynucleotidyl transferase; TUNEL assay) (green), and induction of a DDR detected by 53BP1 foci (red) as marker of the NHEJ pathway

**Figure S1B**. Induction of DNA damage by doxorubicin and triggering a DNA-Damage response (DDR) by the NHEJ pathway in A549 cells.. VRK1 depletion impairs the DDR detected by 53BP1 foci (red) but does not affect the DNA damage detected by TUNEL assay (green) after treatment of A549 cells with doxorubicin. At the bottom is shown an immunoblot to confirm the VRK1 depletion.

hours


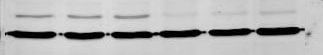


VRK1

β actin

─ 50

─ 37

0 0.5 2 0 0.5 2

siCt

siV-02

Doxo, 10 µM


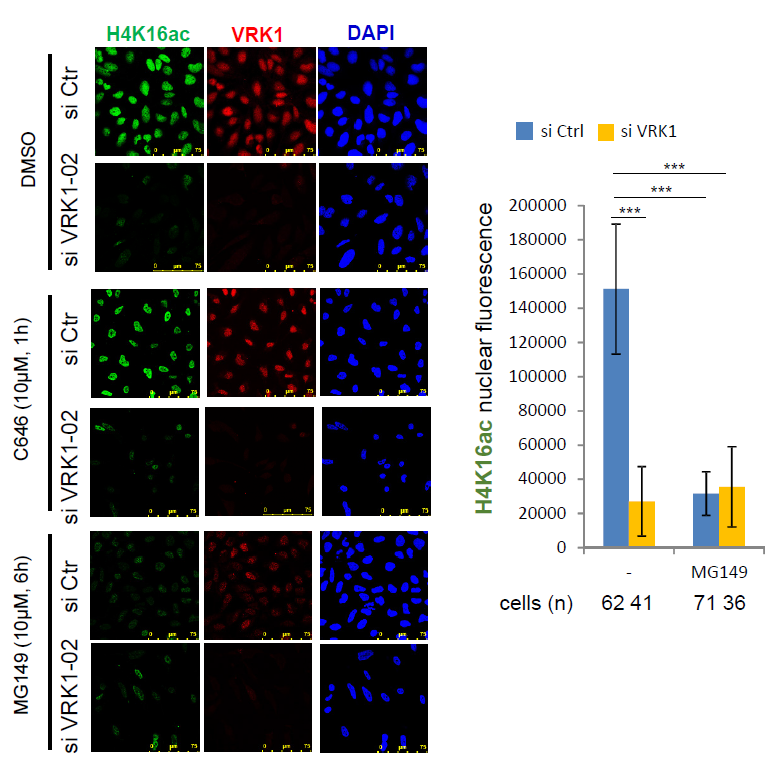


**Figure S2**. Effect of acetyl transferase inhibitors on the basal level of endogenous H4K16 acetylation. U2OS cells in 0.5 % serum were treated with the the p300 inhibitor (C646) or the Tip60 inhibitor (MG149) for the times and concentrations indicated. The level of endogenous H4K16ac and VRK1 were determined by immunofluorescence. The quantification is shown in the graph to the right. *** P < 0.001

**Figure S3**. Phosphorylation of ATM is lost by depletion of VRK1 or inhibition of Tip60 in A549 (ATM+/+) cells. Field images. **A**. Effect of VRK1 depletion orTip60 inhibition on the phosphorylation of ATM in Ser1981. A549 cells were treated with doxorubicin and the effect of VRK1 depletion on the detection of phosphorylated ATM was determined by immunofluorescence with a specific antibody. The selected individual cell is shown in Figure 7A. **B**. Effect of Tip60 inhibition with MG149 on the phosphorylation of ATM induced by doxorubicin and detected by immunofluorescence. The selected individual cell is shown in Figure 7B.

Table S1. Primary antibodies and conditions of applications


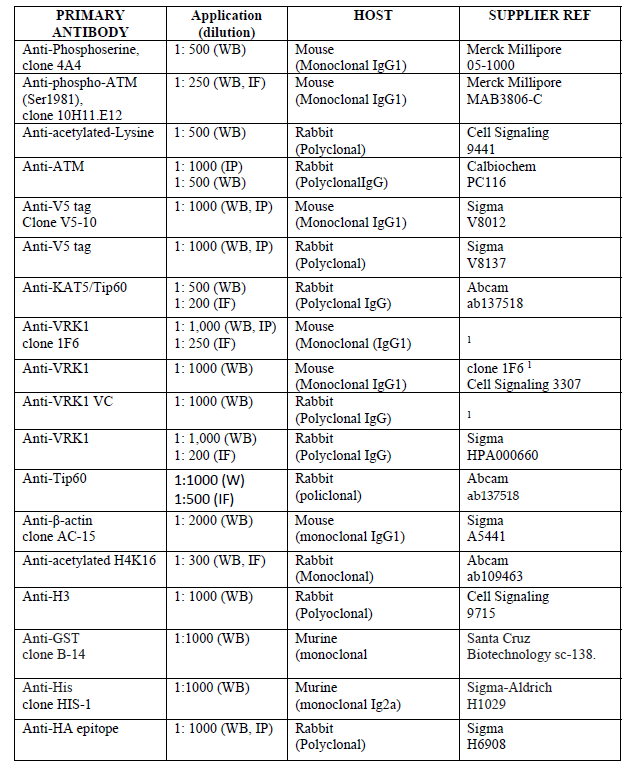


1 Valbuena A, Lopez‐Sanchez I, Vega FM, Sevilla A, Sanz‐Garcia M, Blanco S et al. Identification of a dominant epitope in human vaccinia‐related kinase 1 (VRK1) and detection of different intracellular subpopulations. Arch Biochem Biophys 2007; 465: 219‐226.

Table S2. Secondary antibodies and conditions of applications


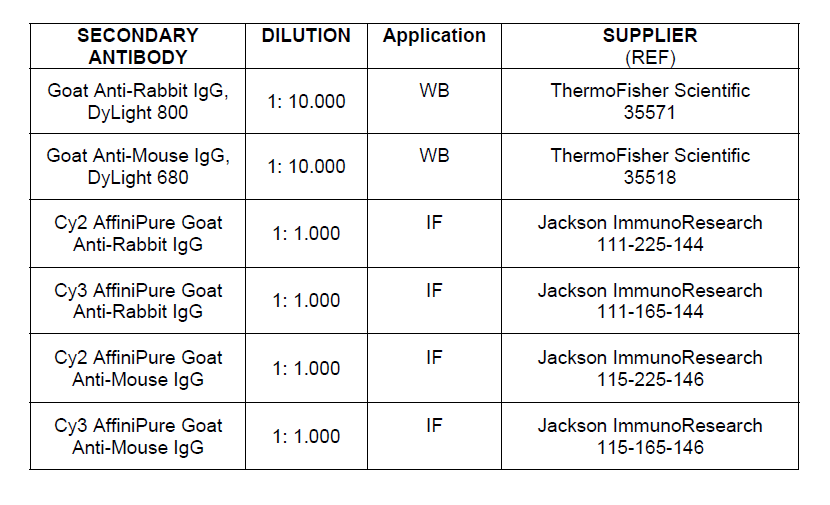

Supplement: Supplementary file 1 [file cancers-12-02986-s001.zip › cancers-918796-supplement.docx]
