# Supplementary material for: VRK1 Phosphorylates Tip60/KAT5 and Is Required for H4K16 Acetylation in Response to DNA Damage
Source: Cancers (Basel). 2020 Oct 15;12(10):2986. doi: 10.3390/cancers12102986 (PMC7650776; doi:10.3390/cancers12102986)

Supplementary file original gels

Fig. 1A

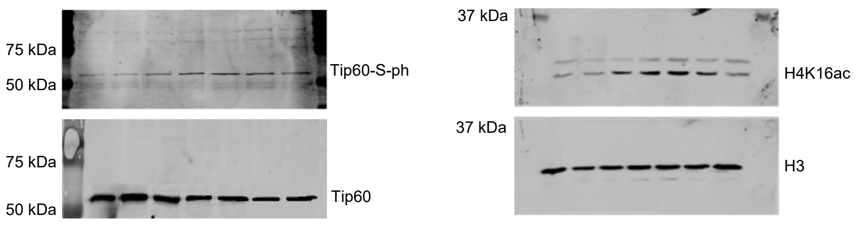

Fig. 2B

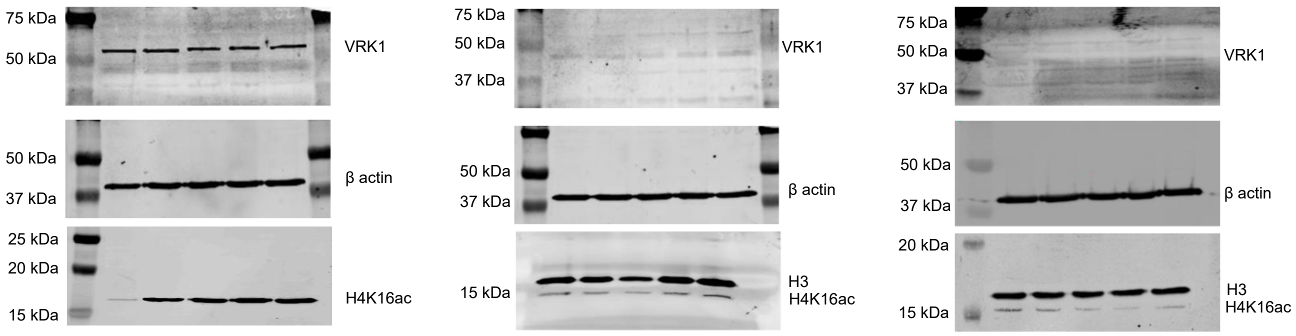

Fig. 2C

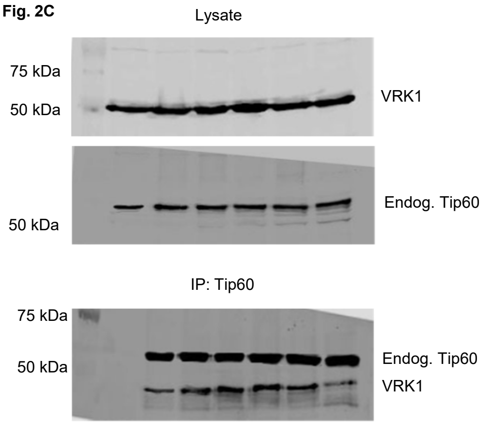

Fig. 3B

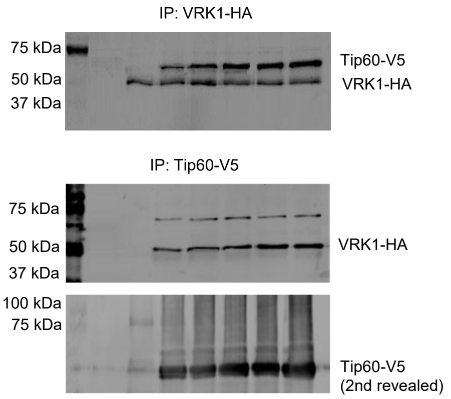

Fig. 4A

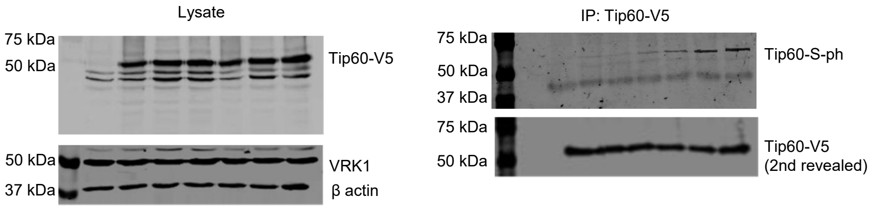

Supplementary file original gels

Fig. 4B

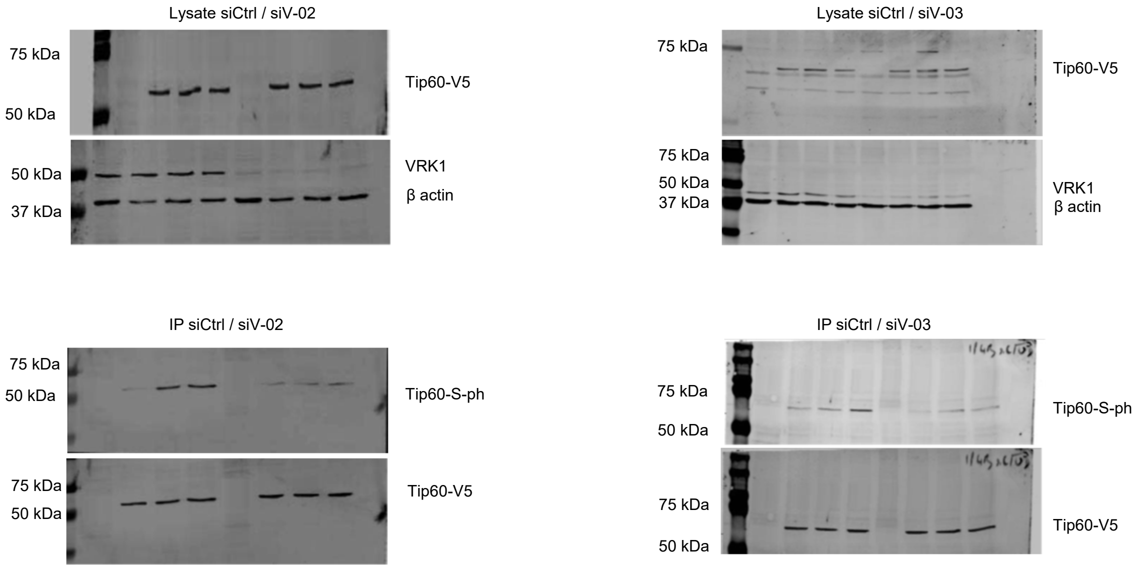

Fig. 4C

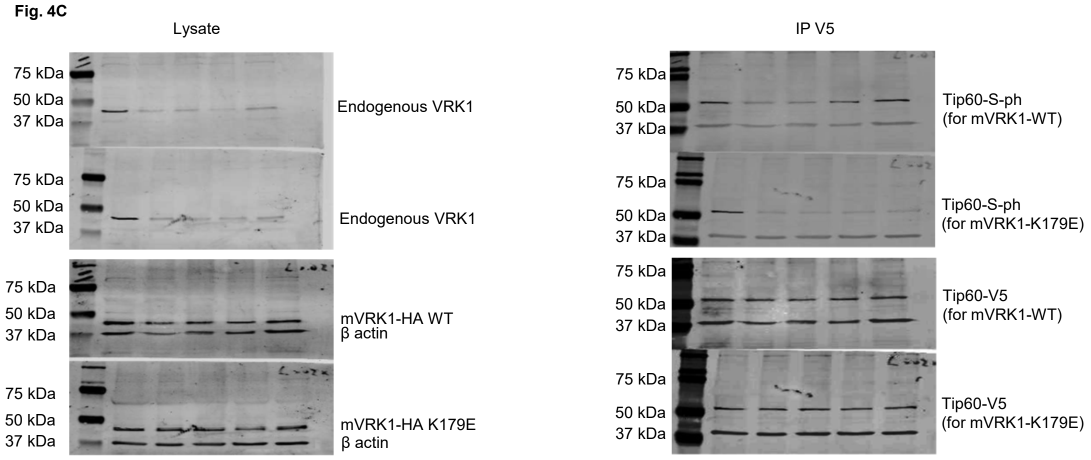

Supplementary file original gels

Fig. 5B

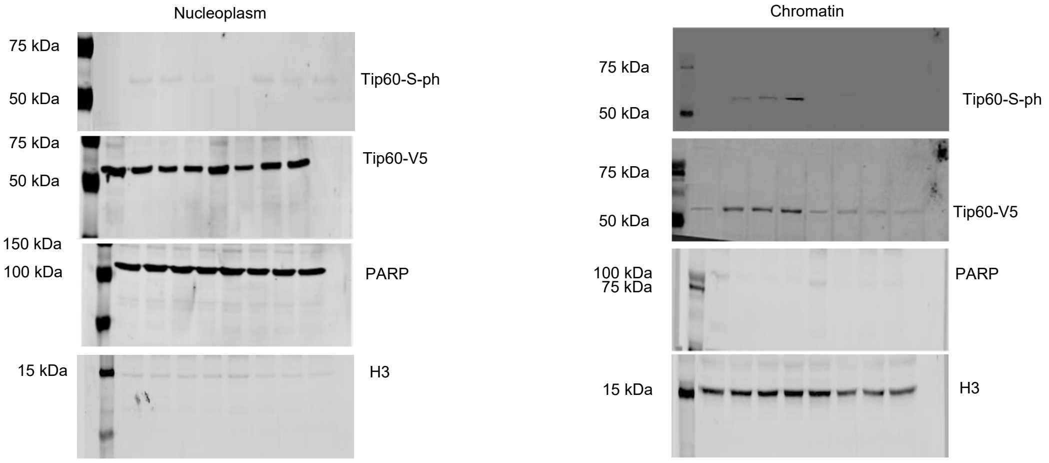

Fig. 6B

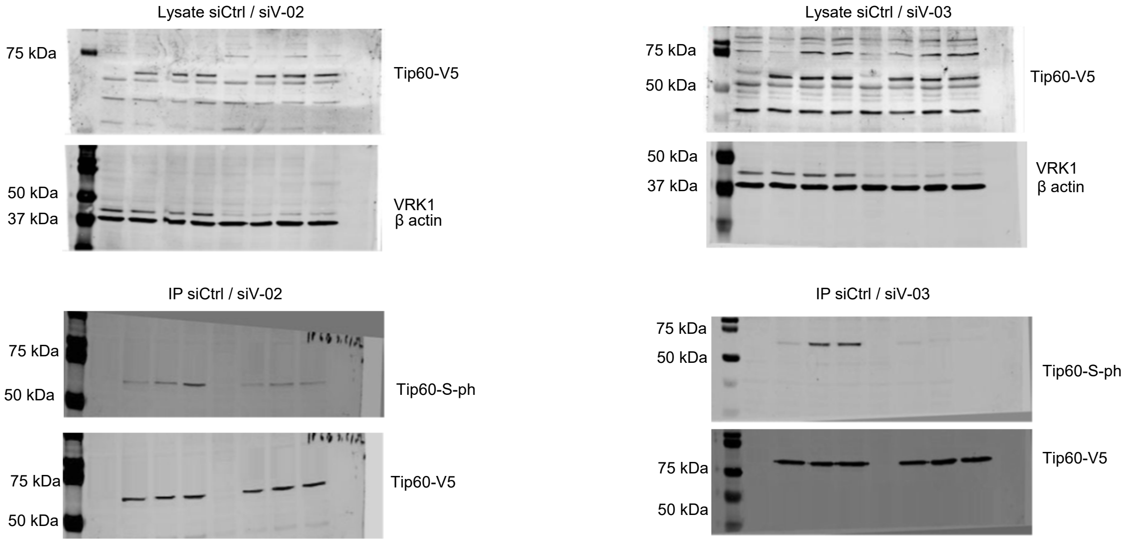

Fig. 7A

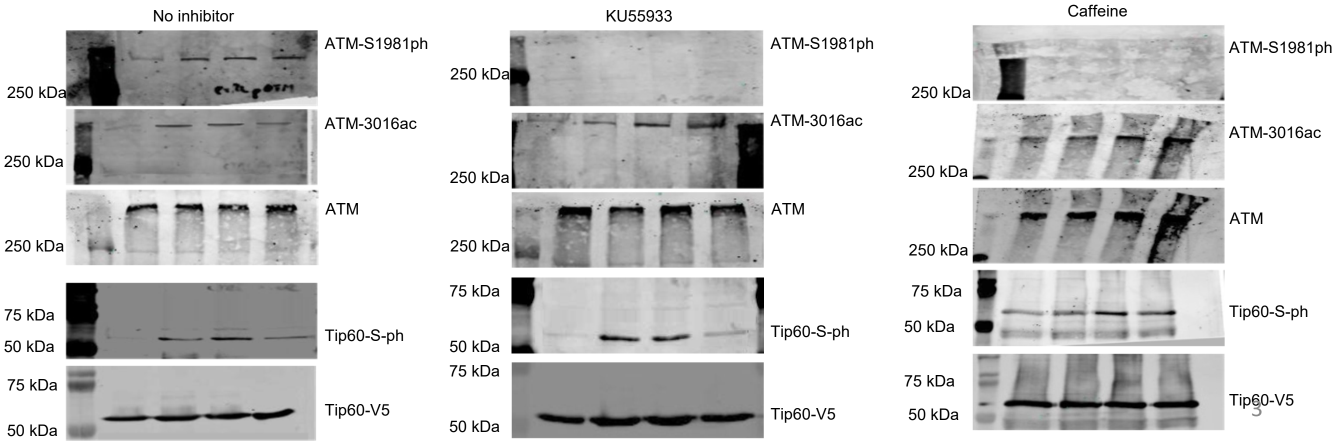

Supplementary file original gels

Fig. 7B

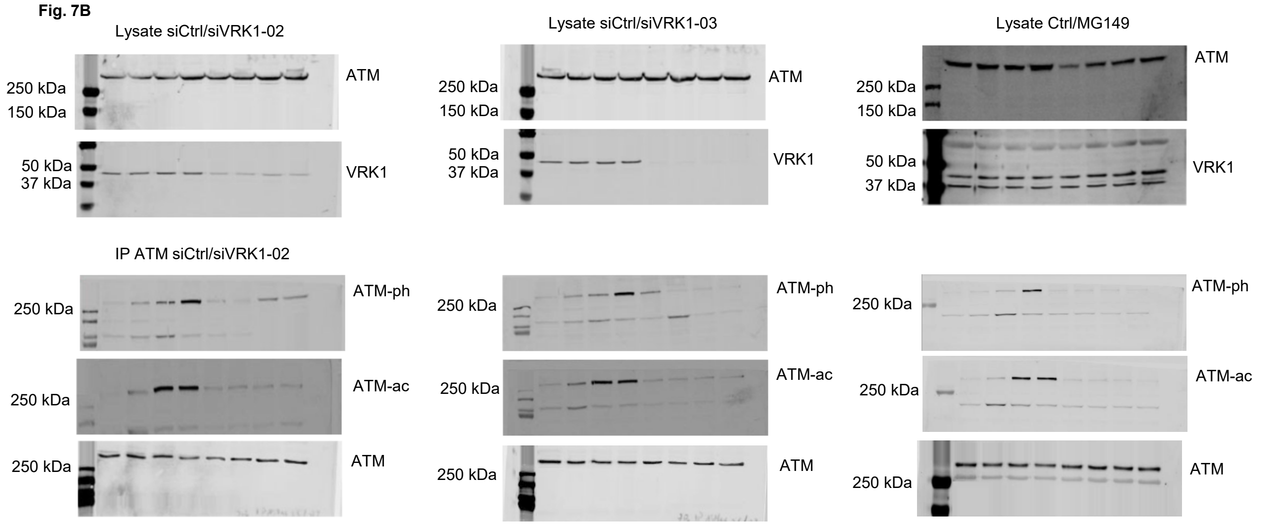

Fig. 7C

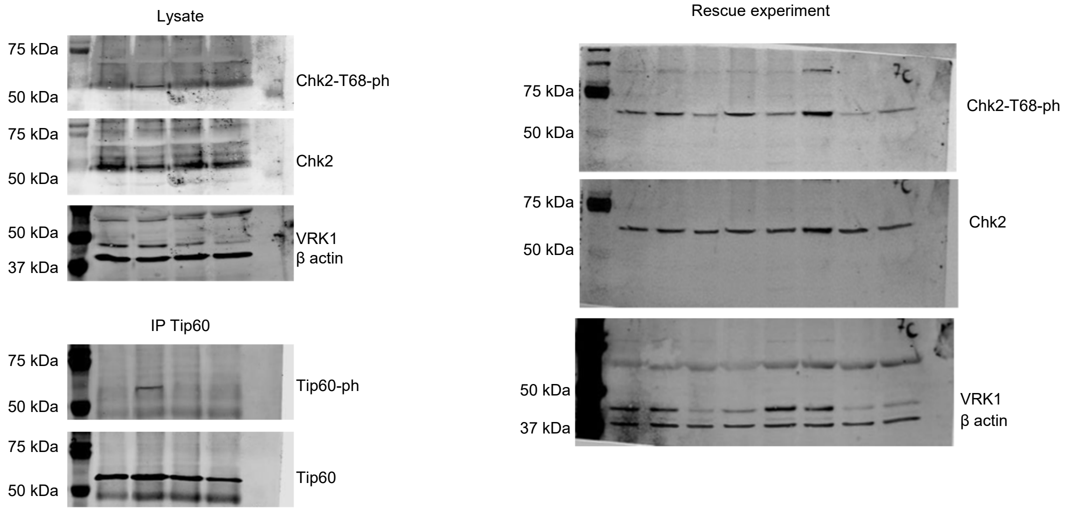

Fig. 9A

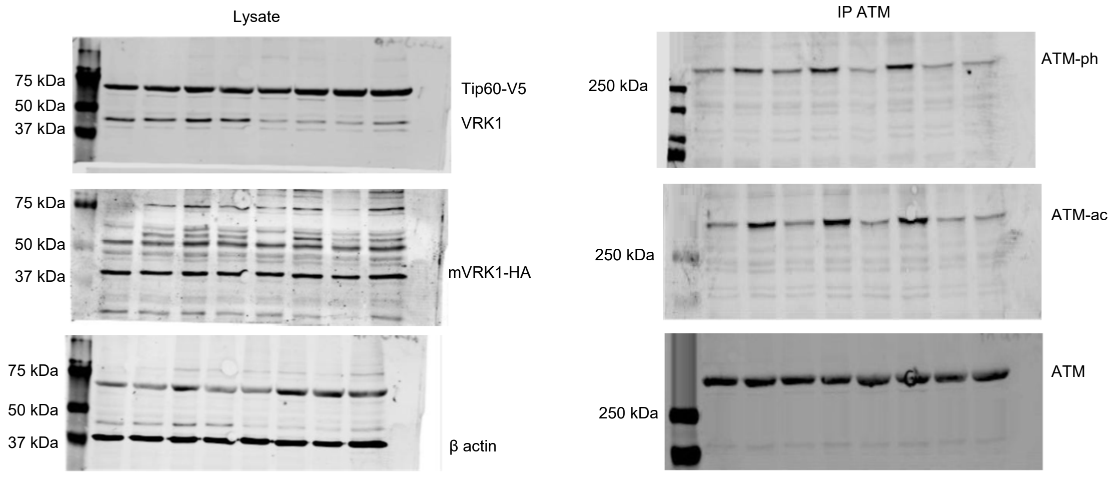

Supplement: Supplementary file 1 [file cancers-12-02986-s001.zip › Suplementary original gels.pdf]
